# MAPK3 as a ferroptosis driver in excitatory neurons in Alzheimer’s disease: from genetic causal inference to *in vivo* functional validation

**DOI:** 10.3389/fnagi.2026.1868908

**Published:** 2026-09-24

**Authors:** Xiaoqiong An, Changge Wei, Manni Cao, Jianwei Xu, Wenfeng Yu, Zhenkui Ren

**Affiliations:** 1 Department of Anatomy, School of Basic Medicine, Guizhou Medical University, Guiyang, Guizhou, China; 2 Key Laboratory of Human Brain Bank for Functions and Diseases of Department of Education of Guizhou Province, Guizhou Medical University, Guiyang, Guizhou, China; 3 Department of Laboratory Medicine, The Second People’s Hospital of Guizhou Province, Guiyang, Guizhou, China; 4 Center for Tissue Engineering and Stem Cell Research, Guizhou Medical University, Guizhou, China; 5 School of Pharmaceutical Sciences, Guizhou Medical University, Guizhou, China; 6 Department of Pharmacology, School of Basic Medicine, Guizhou Medical University, Guizhou, China

**Keywords:** Alzheimer’s disease, eQTL, ferroptosis, MAPK3, mendelian randomization (MR)

## Abstract

**Introduction:**

Alzheimer’s disease (AD) is a progressive neurodegenerative disorder, and ferroptosis-an iron-dependent form of regulated cell death-has been increasingly implicated in AD-related neuronal loss. However, the causal relationship between specific ferroptosis-related genes and AD, particularly within defined neuronal subtypes, remains incompletely understood.

**Methods:**

We integrated two-sample Mendelian Randomization (MR) with single-cell expression quantitative trait locus (eQTL) analysis, followed by in vivo validation using AAV9-c-fos-mediated MAPK3 knockdown in APP/PS1 transgenic mice and in vitro validation using siRNA-mediated MAPK3 silencing in Aβ-treated HT22 neurons.

**Results:**

MR analysis of large-scale genetic datasets identified seven ferroptosis-related genes with causal associations with AD. Single-cell eQTL MR prioritized MAPK3 as a significant risk factor specifically within excitatory neurons. AAV9-c-fos-mediated MAPK3 knockdown in APP/PS1 mice significantly ameliorated cognitive deficits in the Morris water maze. Mechanistically, MAPK3 knockdown inhibited aberrant CaMKII phosphorylation, activated the Nrf2-GPX4 antioxidant axis (upregulation of GPX4, HO-1, and FTH1; downregulation of ACSL4 and KEAP1), and reestablished the Bax/Bcl-2 equilibrium, thereby reducing both ferroptosis and apoptosis. These effects were reproduced in Aβ-treated HT22 neurons following siRNA-mediated MAPK3 silencing.

**Discussion:**

Our findings identify MAPK3 in excitatory neurons as a potential causal link between genetic risk and AD pathogenesis, bridging population-level genetic risk and cellular vulnerability. Targeting the MAPK3-CaMKII-Nrf2 axis may represent a promising, cell-type-specific therapeutic strategy for AD.

## Graphical Abstract

**Figure:**
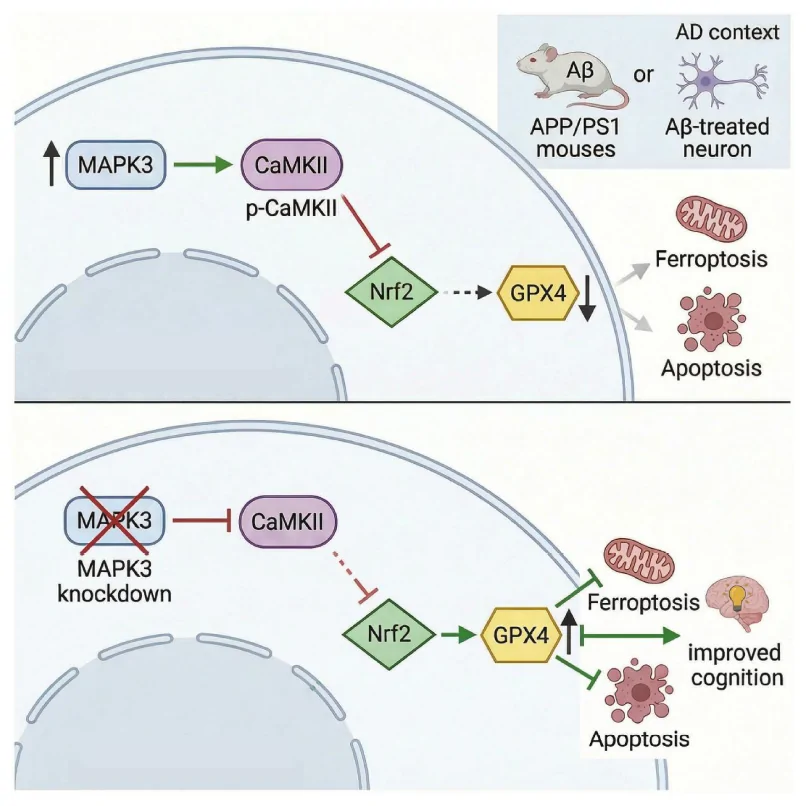
Mechanism of MAPK3 in excitatory neurons linking ferroptosis and apoptosis to Alzheimer’s disease. Upper panel: in the AD context (APP/PS1 mice or Aβ-treated neurons), MAPK3 activates CaMKII through phosphorylation, which suppresses the Nrf2–GPX4 antioxidant axis, leading to ferroptosis and apoptosis. Lower panel: AAV9-c-fos-mediated knockdown of MAPK3 preserves CaMKII homeostasis, restores Nrf2–GPX4 signaling, reduces ferroptosis and apoptosis, and improves cognition.

## Introduction

AD is a major global health issue, marked by a gradual decline in cognitive abilities and loss of neurons (Breijyeh and Karaman, 2020; Rajendran and Krishnan, 2024). Its development is complex, involving the buildup of amyloid-β (Aβ) plaques, neurofibrillary tangles of hyperphosphorylated tau, synaptic issues, and various types of programmed cell death (Zhang et al., 2024; Zheng and Wang, 2025). Among these, ferroptosis—an iron-dependent form of regulated cell death caused by lipid peroxidation—has recently been identified as a key factor in AD-related neurodegeneration (Yang and Nao, 2022; Zhang et al., 2024). This type of cell death is closely controlled by important pathways, such as the nuclear factor erythroid 2-related factor 2 (Nrf2) and glutathione peroxidase 4 (GPX4) axis, which act as vital cellular defenses against oxidative damage and ferroptosis (Cao et al., 2025; Yang et al., 2022; Yuan et al., 2021). Despite increasing awareness of its significance, the exact causal relationships between specific ferroptosis-related genes and AD risk, especially in certain neuronal groups, are not well understood.

Recent investigations into the pathogenesis of AD have increasingly concentrated on the dysregulation of specific signaling kinases and neuronal excitability. Notably, the mitogen-activated protein kinase (MAPK) pathways, including p38α MAPK, have been implicated in neuroinflammation and tau pathology (Falcicchia et al., 2020; Xue et al., 2024; Zoatier et al., 2025). Additionally, calcium/calmodulin-dependent kinases, such as CaMKII and death-associated protein kinase 1 (DAPK1), play crucial roles in mediating synaptic plasticity, excitotoxicity, and neuronal apoptosis in response to amyloid-beta (Aβ) oligomers (Chan et al., 2022; Xu et al., 2019, 2023). However, the majority of research has either focused on broad genetic associations or isolated molecular pathways, often lacking causal inference. Although genome-wide association studies (GWAS) have identified numerous risk loci, a significant gap persists in translating these statistical associations into a mechanistic understanding at the cell-type-specific level of how genetic variants—especially those linked to ferroptosis—contribute to AD risk.

The primary methodological advancement is the utilization of Two-Sample MR analysis, an advanced epidemiological technique that employs genetic variants as instrumental variables to deduce causal relationships between gene expression and disease risk (Gkatzionis et al., 2023; Shapland et al., 2019). This method effectively addresses the major limitations of observational studies by reducing confounding factors and reverse causation (Lovegrove et al., 2024). A notable strength of our approach is the sequential application of MR: initially employing bulk tissue expression quantitative trait loci (eQTL) data to identify candidate ferroptosis genes potentially linked to AD, followed by single-cell eQTL MR analysis. This latter phase is essential for determining the specific brain cell type—such as excitatory neurons, inhibitory neurons, or glial cells—through which a causal gene exerts its influence, thereby providing a crucial layer of cellular specificity that is often absent in genetic studies (Yu et al., 2022).

Building on genetic discoveries, this study employs rigorous functional validation across complementary model systems to establish biological plausibility and elucidate mechanisms. *In vivo*, we utilize an adeno-associated virus (AAV) delivery system with an excitatory neuron promoter (c-fos) to achieve targeted knockdown of a key candidate gene in the APP/PS1 transgenic mouse model of AD. This approach facilitates the investigation of gene function within a specific, disease-relevant neuronal population, thereby reducing the confounding effects associated with global manipulation (Case et al., 2024; Qu et al., 2025). *In vitro*, hippocampal neuronal cells are employed to dissect downstream signaling pathways. This multi-tiered validation framework—from genetics to controlled cellular and animal models—ensures that the observed effects are robust, reproducible, and directly pertinent to the hypothesized pathophysiology of AD.

This study will be conducted from three distinct perspectives. First, it aims to systematically identify and validate ferroptosis-related genes that demonstrate a causal relationship with the risk of AD through the application of MR methodology. Second, the research seeks to determine the specific brain cell type that mediates the effects of a top-ranked causal gene, thereby establishing a connection between genetic risk and cellular vulnerability. Third, the study endeavors to functionally characterize the role of this gene in AD-related phenotypes, including cognitive deficits, and the activation of cell death pathways such as ferroptosis and apoptosis. By integrating causal inference derived from human genetics with mechanistic analysis in experimental models, this research aims to elucidate a novel pathway linking genetic susceptibility, neuronal subtype dysfunction, and ferroptotic cell death in AD, ultimately identifying precise therapeutic targets.

## Materials and methods

### Data source

Extracting key genes related to ferroptosis from public databases FerrDb^1^. which included 483 genes. All eQTL data for the genes in this study were downloaded from the eQTLGen database^2^ (Võsa et al., 2021). All data are cis-eQTL, derived from blood samples. We screened instrumental variables (SNP) according to the following parameters: *p*-value < 5e-8, clumping window: 10000 kb, r2 < 0.1, and finally, there are 15695 genes with cis-eQTL data remaining. The intersection of the above genes with 483 ferroptosis genes resulted in 315 ferroptosis genes with cis-eQTL data. Genome-wide association study (GWAS) data for AD were obtained from the FINNGEN database^3^. This dataset comprised a total of 500,348 participants, including 13,964 AD cases and 486,384 controls. We obtained AD transcriptome data from the GEO database, specifically dataset GSE132903 with 97 AD and 98 control samples. All samples were normalized for consistency. Using the “limma” package, we identified differentially expressed genes (DEGs) between AD and control samples, applying a threshold of p < 0.05 and |log2FC| > 0.25.

### Two-sample mendelian randomization (MR) links ferroptosis genes to AD

To assess the causal effects of ferroptosis-related genes on AD, a two-sample MR analysis was performed using the TwoSampleMR package. A total of 315 ferroptosis genes were utilized as exposure variables, with AD designated as the outcome variable. To mitigate bias arising from weak instruments, single nucleotide polymorphisms (SNPs) with an F-statistic below 10 were excluded prior to conducting the MR analyses. The inverse variance weighted (IVW) method was employed as the primary analytical approach, complemented by the weighted median and MR-Egger regression as sensitivity analyses. To account for multiple testing across the 315 candidate genes, the false discovery rate (FDR) was controlled using the Benjamini-Hochberg procedure. The robustness of the genetic associations was further assessed by testing for heterogeneity (Cochran’s Q) and horizontal pleiotropy (MR-Egger intercept), together with leave-one-out analysis. Results were deemed statistically significant when the *P*-value of the IVW analysis was less than 0.05. Our analysis identified a significant association between 47 of the ferroptosis genes and AD.

### EQTL-based two-sample MR validation links seven ferroptosis genes to AD

To further verify the accuracy of the above results, 47 ferroptosis genes eQTL, derived from blood samples in GTEx database^4^. Instrumental variables SNPs were screened, according to the following parameters: *p*-value < 5e-8, clumping window: 10000 kb, r2 < 0.1, and finally, there are 34 ferroptosis genes with eQTL data remaining. To evaluate the causal impact of 34 ferroptosis genes on AD, a two-sample MR analysis was conducted using the TwoSampleMR package. The genes served as exposure variables, and AD was the outcome variable. SNPs with an F-statistic below 10 were excluded to reduce bias from weak instruments. The IVW method was the main analysis tool, complemented by the weighted median and MR-Egger methods as sensitivity analyses, with multiple testing controlled using the Benjamini-Hochberg FDR procedure. Results were considered significant at a *P*-value below 0.05. The analysis found a significant link between 7 ferroptosis genes and AD.

### Single-cell eQTL MR reveals MAPK3’s causal role in excitatory neurons for AD

To assess the potential causal relationship between 7 ferroptosis-related genes and AD in the context of eight distinct brain cell types. Single-cell expression quantitative trait loci (sceQTL) data for the SESN2, VCP, GCH1, MAPK3, MAP1LC3A, RPL8 and ACSF2 genes were sourced from the scQTLbase database^5^ (Ding et al., 2024). The dataset included single-cell eQTL information across eight distinct brain cell types: astrocytes, endothelial cells, excitatory neurons, inhibitory neurons, microglia, oligodendrocytes, oligodendrocyte precursor cells, and pericytes. Instrumental variables, specifically SNPs, were selected based on a *p*-value threshold of less than 0.05, with clumping parameters set at clump_kb = 100 and clump_r2 = 0.1, and finally, there are 4 ferroptosis genes (VCP, MAPK3, MAP1LC3A, SESN2) with sceQTL data remaining.

To assess the potential causal relationship between 4 ferroptosis-related genes and AD in the context of brain cell, a two-sample MR analysis was performed utilizing the TwoSampleMR package. In this analysis, the ferroptosis genes were treated as exposure variables, while AD was designated as the outcome variable. SNPs with an F-statistic less than 10 were excluded to mitigate bias from weak instruments. The weighted median method was employed as the primary analytical approach, with statistical significance determined at a *P*-value threshold of less than 0.05. The analysis revealed a significant positive association between the MAPK3 gene and excitatory neurons.

### Antibodies and reagents

Aβ_1–42_ peptide ( > 95% purity) was chemically synthesized by Shanghai Qiangyao Biotechnology (Shanghai, China). Anti-MAPK3 (1:1000, Cat# A75469), Anti-GPX4 (1:1000, Cat# A74526), Anti-ACSL4 (1:1000, Cat# A95060), Anti-Nrf2 (1:1000, Cat# A42970), Anti-Keap1 (1:1000, Cat# A91657), Anti-HO-1 (1:1000, Cat# A24078), and Anti-FTH1 (1:1000, Cat# A46681) antibodies were purchased from Nature Biosciences (Hangzhou, China). Anti-CaMKII (1:1000, Cat# ET1704-37), Anti-P-CaMKII (1:1000, Cat# HA721794), Anti-XCT (1:1000, Cat# HA724114), Anti- BAX (1:1000, Cat# ET1603-34), and Anti-BCL2

(1:1000, Cat#HA723111) antibodies were purchased from HuaAn Biotechnology Co., Ltd., (Hangzhou, China). NCOA4 (1:1000, Cat# A5695) was purchased from ABclonal Technology (Wuhan, China). GAPDH (1:2000, Cat# 60004-1-Ig; Proteintech) was purchased from Proteintech (Wuhan, China).

### Cell culture and cell treatment

HT22 mouse hippocampal neuronal cells were obtained from Wuhan Procell Biotechnology Co., Ltd., (Wuhan, China) and were cultured in Dulbecco’s Modified Eagle Medium (DMEM) supplemented with 10% fetal bovine serum (FBS) and 1% penicillin-streptomycin at 37°C in a humidified incubator with 5% CO_2_. The cells were subcultured at 70–80% confluence. For experiments, the cells were treated with 1 μM Aβ_1–42_ oligomers for 24 h.

RNA interference (RNAi) of MAPK3For RNA interference experiments, HT22 cells were seeded at 5 × 10^5^ cells/mL in 6-well plates and cultured for 24 h to reach 60–70% confluence. The cells were then transfected with 50 nM MAPK3 siRNA or negative control siRNA (Hanheng Biotechnology, Shanghai, China) using Lipofectamine 3000 (Invitrogen, USA) according to the manufacturer’s instructions. At 24 h post-transfection, cells were treated with 1 μM aggregated Aβ_1–42_ for an additional 24 h. Cells were then harvested for Western blot analyses. Knockdown efficiency was confirmed by Western blot at 48 h post-transfection. The siRNA sequences used for MAPK3 knockdown were as follows: si-MAPK3, sense 5′-CUUCCAAUCUGCUUAUCAATT-3′ and antisense 5′- UUGAUAAGCAGAUUGGAAGTT-3′; negative control siRNA (si-NC), sense 5′- UUCUCCGAACGUGUCACGUTT, and antisense 5′- ACGUGACACGUUCGGAGAATT-3′. All transfection experiments were performed in three independent biological replicates.

### Preparation of amyloid

Lyophilized Aβ_1–42_ was dissolved in hexafluoroisopropanol (HFIP) to a final concentration of 1 mM. The solution was incubated at room temperature for 60 min, then placed on ice for 10 min, followed by evaporation of HFIP in a fume hood to form a transparent peptide film. The film was resolubilized in anhydrous DMSO (5 mM stock), diluted to 100 μM in serum-free DMEM, and incubated at 4°C for 24 h to allow oligomerization. Following incubation, the solution was centrifuged at 14,000 × *g* for 10 min at 4°C to remove insoluble aggregates, and the supernatant containing soluble Aβ oligomers was collected for cell treatment.

### Animals and experimental design

Six-month-old male APP/PS1 double-transgenic Alzheimer’s disease (AD) mice and age-matched C57BL/6 wild-type (WT) mice were obtained from were obtained from SLAC Laboratory Animal Co., Ltd., in Shanghai, China. Animals were randomly assigned to four experimental groups: WT, APP/PS1, APP/PS1-AAV-NC, and APP/PS1-AAV-shMAPK3. All mice were housed under standardized conditions (22 ± 2°C, 50–60% humidity, 12-h light/dark cycle, ventilation rate 8–15 air changes/h) with *ad libitum* access to food and water. Following the behavioral tests, mice were deeply anesthetized with an intraperitoneal injection of pentobarbital sodium (100 mg/kg body weight). Once fully anesthetized and unresponsive to stimuli, they were euthanized by cervical dislocation. The brains were promptly extracted for further molecular and histological examination. The animal experiments conducted in this study received ethical approval from the Animal Care and Use Committee of the Second People’s Hospital of Guizhou Province (permit number: 202102) and were performed in compliance with the ARRIVE 2.0 guidelines.

### Intracerebroventricular injection

MAPK3 was silenced using an AAV vector delivery system. A short hairpin RNA (shRNA) targeting MAPK3, an enhanced green fuorescent protein (EGFP) reporter gene, and the neuron-specifc c-fos promoter were synthesized and cloned into AAV packaging vectors. For intracerebroventricular injection, mice were anesthetized with isoflurane (induction 3–5%, maintenance 0.5–2%) and secured in a stereotaxic frame. After a midline scalp incision to expose the skull, a total of 3 μL of AAV-sh-NC or AAV-sh-MAPK3 (viral titer: 1 × 10^1^2^^ vg/mL) was infused into the right lateral ventricle at a rate of 0.2 μL/min via stereotaxic injection using a microsyringe pump, following established protocols. The needle was left in place for 5 min post-injection to ensure adequate diffusion before withdrawal, and the incision was sutured. The mice were kept on a heating pad until they recovered. Four weeks after the injection, behavioral assessments were conducted. The mice were then euthanized, and brain tissues were collected for molecular and morphological analyses.

### Morris water maze test

The Morris water maze test, a widely used paradigm for assessing spatial learning and memory, was conducted in a circular stainless steel pool (150 cm in diameter, 60 cm in height) filled with water rendered opaque by the addition of titanium dioxide powder (22 ± 1°C). The pool was conceptually divided into four equal quadrants, with distinct visual cues affixed to the wall of each quadrant. A hidden platform (10 cm in diameter) was placed 1 cm below the water surface at the center of the target quadrant. The procedure consisted of three phases: adaptation, training, and probe trial. During the adaptation phase, mice were allowed to swim freely for 60 s without a platform to acclimate to the pool. In the training phase, mice were placed into the water facing the pool wall from three different starting points and given 60 s to locate the hidden platform. The time taken to find and climb onto the platform was recorded as escape latency. If a mouse failed to find the platform within 60 s, it was gently guided to it and allowed to remain there for 20 s. Training was performed for five consecutive days with three trials per day. 24 h after the last training session, the probe test was conducted to evaluate spatial memory retention. The platform was removed from the pool, and each mouse was allowed to swim freely for 60 s. The time spent in the target quadrant, the number of crossings over the former platform location, and the swimming trajectory were recorded and analyzed. All trials were recorded and analyzed using the ANY-maze video tracking system.

### Western blot

Total protein was extracted from mouse brain tissues and HT22 cells using RIPA lysis buffer containing protease inhibitors. Tissues were homogenized in a grinder at 4°C, while cells were directly lysed by sonication on ice for 30 min. All samples were then centrifuged at 12,000 rpm for 15 min at 4°C, and the supernatant was collected. Protein concentrations were determined using a BCA assay kit. Equal amounts of protein samples were separated by SDS-PAGE, transferred onto polyvinylidene fluoride (PVDF) membranes, and blocked with 5% non-fat milk for 2 h at room temperature. After blocking, the membranes were incubated overnight at 4°C with primary antibodies. After incubation, the primary antibody was collected and stored for future use. The membranes were then washed three times with TBST (8 min each), followed by incubation with HRP-conjugated secondary antibody (1:1000) for 2 h at room temperature. After three additional washes with TBST, protein bands were visualized using enhanced chemiluminescence (ECL) reagent and analyzed with Image J software.

### Immunofluorescence

Mouse brain sections were fixed with 4% paraformaldehyde for 15 min at room temperature, washed with PBS, permeabilized with 0.5% Triton X-100 for 15 min, and blocked with 10% goat serum for 60 min. The sections were then incubated overnight at 4°C with primary antibodies against c-fos (1:200; Affinity), ACSL4 (1:400; Abcam). After three washes with PBS, the sections were incubated with goat anti-rabbit IgG secondary antibody (1:1000; ab150077, Abcam) for 1 h at room temperature, followed by three additional PBS washes. Nuclei were counterstained with DAPI (Beyotime, China). Fluorescence images were captured using a fluorescence microscope.

### Statistical analysis

Data were analyzed using GraphPad Prism 9.0 and expressed as mean ± SD. Comparisons between two groups were performed with Student’s *t*-test, while multiple group comparisons were assessed by one-way or two-way ANOVA followed by Tukey’s *post hoc* test. Statistical significance was defined as *p* < 0.05.

## Results

### MR analysis identifies excitatory neuron MAPK3 as a causal ferroptosis gene in AD

This cross-analysis identified 315 ferroptosis genes with available cis-eQTL data from an initial set of 483 (Figure 1A). Using two-sample MR, 47 of these ferroptosis genes showed a potential causal association with AD. Further validation with blood-derived eQTL data from the GTEx database refined this association, confirming a significant causal link for 7 specific genes (Figure 1B); this result was primarily based on the inverse variance weighted (IVW) method, with significance set at *P* < 0.05 (Figure 1C). To investigate cell-type-specific mechanisms, a single-cell eQTL MR analysis was performed. It revealed that among the candidate genes, MAPK3 could promote AD risk specifically through excitatory neurons (Figure 1D). Consistently, MAPK3 was found to be highly expressed in AD contexts (Figure 1E).

**FIGURE 1:**
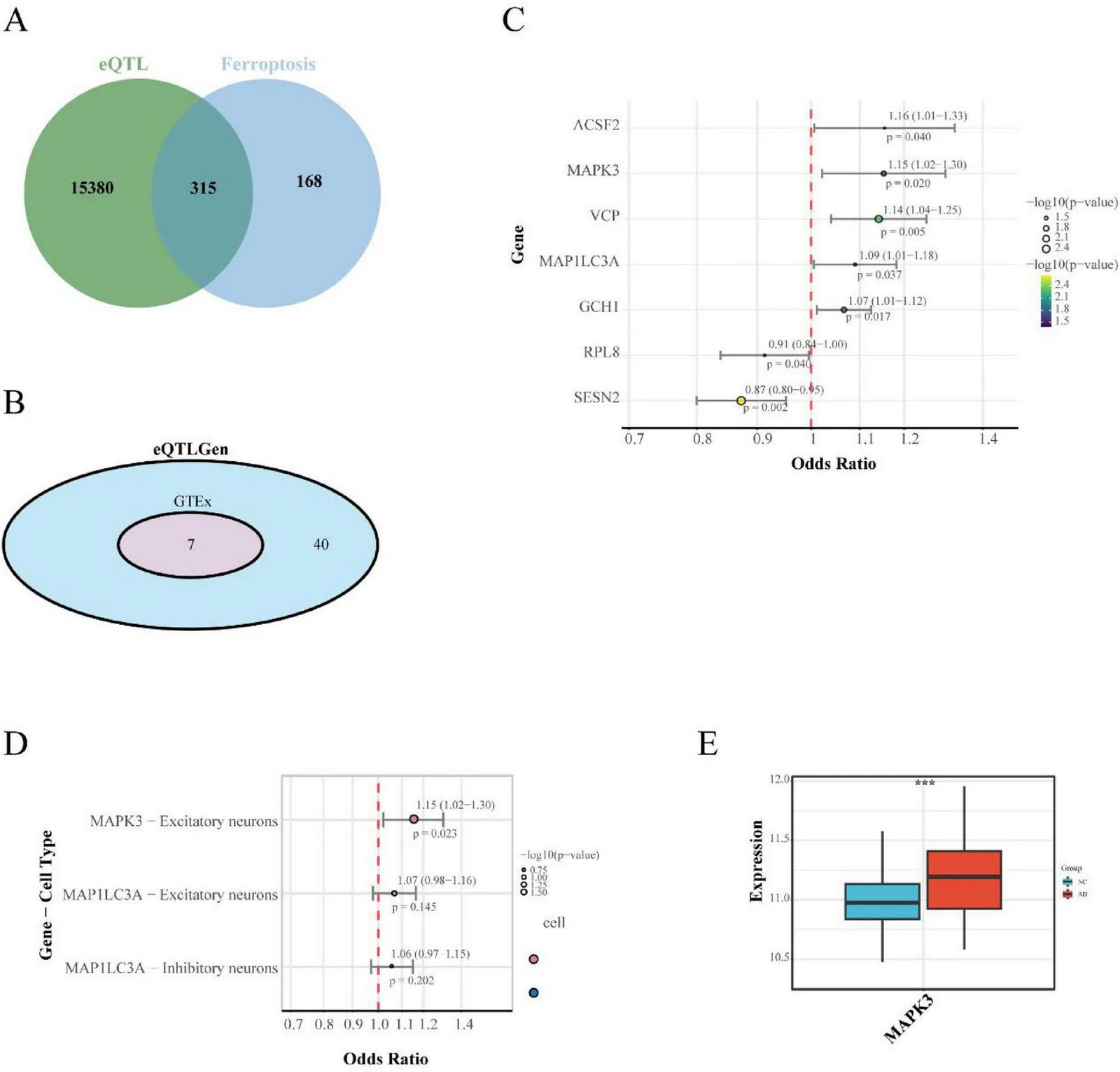
MAPK3 in excitatory neurons links ferroptosis to AD risk. **(A)** Cross-analysis showed that 315 of 483 ferroptosis genes overlapped with those having cis-eQTL data. **(B)** Two-sample MR analysis identified 47 ferroptosis genes causally linked to AD, validation using GTEx eQTL data confirmed significant causal associations for seven specific genes. **(C)** The primary IVW analysis (*P* < 0.05) linked these seven genes to AD. **(D)** Single-cell eQTL MR revealed that MAPK3 may promote AD via excitatory neurons. **(D)** MAPK3 is highly expressed in AD **(E)**. Data are expressed as mean ± SD, *p < 0.05, **p < 0.01, ***p < 0.001.

### AAV9-c-fos-mediated MAPK3 knockdown rescues cognitive deficits in APP/PS1 mice.

To elucidate the role of MAPK3 in AD pathogenesis, we developed an AAV9-c-fos system for MAPK3 knockdown and delivered it into 6-month-old APP/PS1 and WT mice. Spatial learning and memory were assessed using the Morris water maze test. During training, APP/PS1 mice exhibited significantly longer escape latencies compared to WT mice, confirming impaired spatial learning (Figures 2A, B). Notably, AAV9-c-fos-mediated MAPK3 knockdown (APP/PS1 +AAV-ShMAPK3) markedly reduced escape latencies relative to the untreated APP/PS1 group, whereas control AAV injection (APP/PS1 +AAV-NC) had no effect (Figures 2A, B). In the probe trial, APP/PS1 mice showed fewer platform crossings, reduced distance traveled, and less time spent in the target quadrant (Figures 2C–E). These deficits were unaffected by AAV-NC administration but were significantly ameliorated by AAV9-c-fos-mediated MAPK3 knockdown (Figures 2C–E). No significant differences in swimming speed were observed among groups, ruling out motor deficits as a confounding factor (Figure 2F). Collectively, these results demonstrate that AAV9-c-fos-mediated MAPK3 knockdown rescues learning and memory deficits in APP/PS1 mice.

**FIGURE 2:**
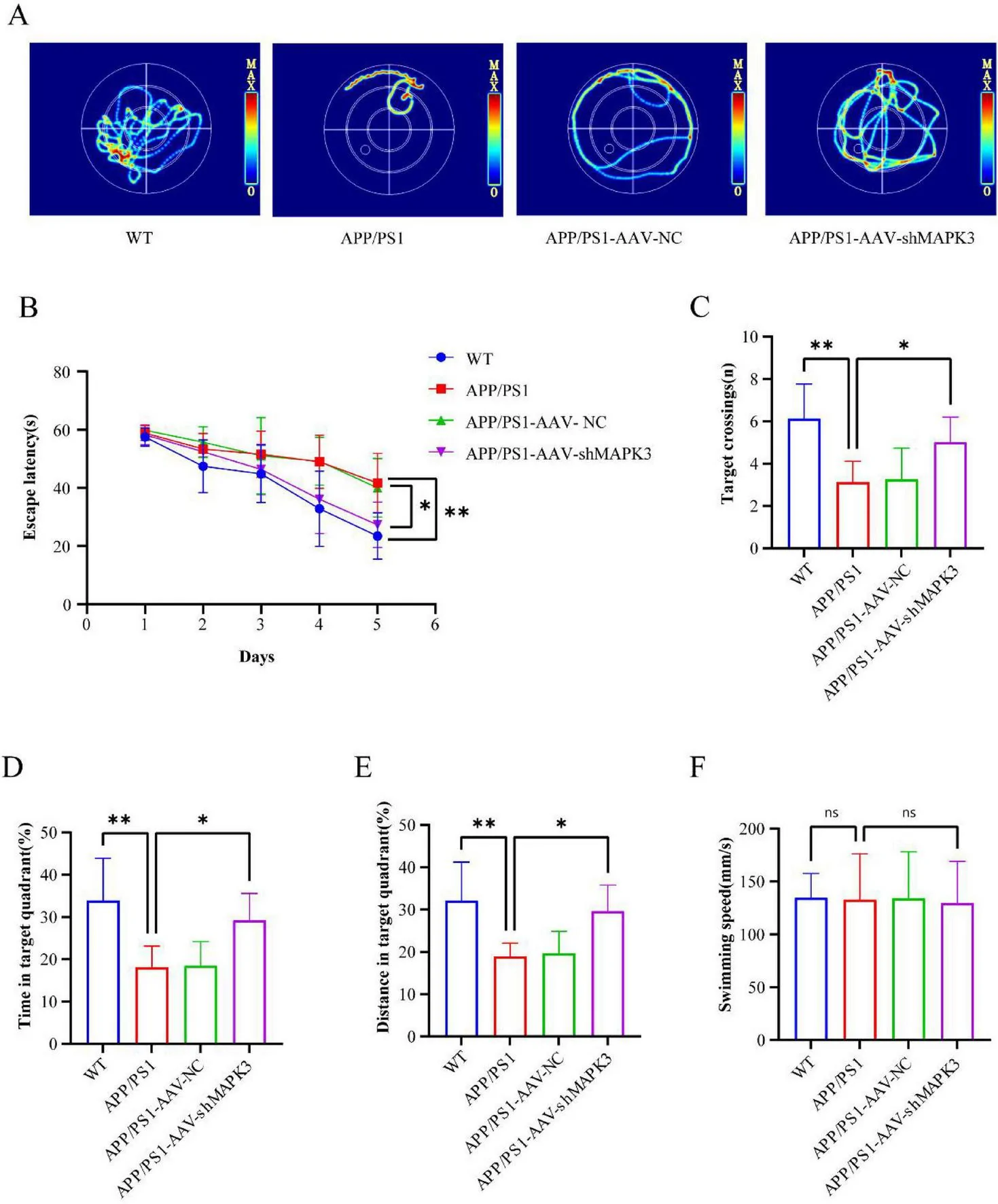
AAV9-c-fos-mediated MAPK3 knockdown improves learning and memory in APP/PS1 mice. **(A)** Representative swimming paths during probe trial. **(B)** Mean escape latency in the Morris water maze experiment of the mice (*n* = 8). **(C)** Number of platform crossings in the probe trial (*n* = 8). **(D)** Distance traveled in the target quadrant (*n* = 8). **(E)** Time spent in the target quadrant (*n* = 8). **(F)** Average swimming speed during the probe trial (*n* = 8). Data are expressed as mean ± SD, *p < 0.05, **p < 0.01, by one-way ANOVA followed by Tukey’s multiple comparisons test.

### AAV9-c-fos-mediated MAPK3 knockdown suppresses aberrant CaMKII activation in APP/PS1 mice

To investigate the downstream mechanisms by which AAV9-c-fos-mediated MAPK3 knockdown ameliorates cognitive deficits in APP/PS1 mice, we first validated the knockdown efficiency of AAV-shMAPK3. Western blot analysis confirmed a significant reduction in MAPK3 protein levels in the APP/PS1+AAV-shMAPK3 group compared to AAV-NC controls (Figure 3A). Based on this finding, we examined the expression and activating phosphorylation of CaMKII, a canonical marker of excitatory neurons (Nicoll and Schulman, 2023). Western blot analysis showed that while total CaMKII protein levels remained unchanged, phosphorylation at Thr286 was significantly reduced in the APP/PS1+AAV-shMAPK3 group compared to untreated APP/PS1 mice (Figures 3B, C). These results indicate that AAV9-c-fos-mediated MAPK3 knockdown suppresses aberrant CaMKII activation.

**FIGURE 3:**
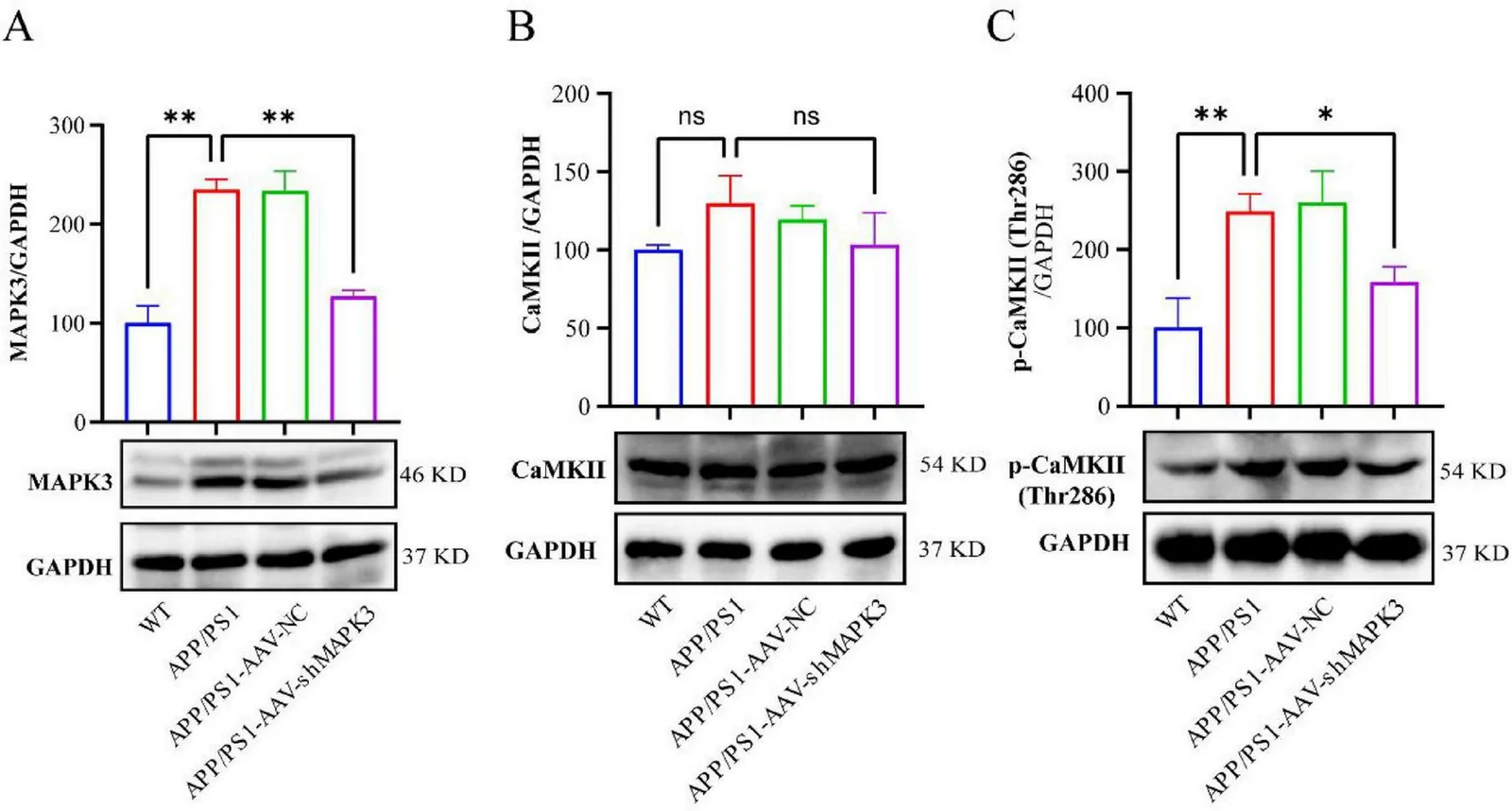
AAV9-c-fos-mediated MAPK3 knockdown inhibits CaMKII phosphorylation. **(A)** Western blot analysis confirming efficient MAPK3 knockdown. **(B)** Representative Western blot images showing total CaMKII levels in each experimental group (*n* = 3). **(C)** Representative Western blot images showing p-CaMKII levels in each experimental group (*n* = 3). Data are expressed as mean ± SD, *p < 0.05, **p < 0.01, by one-way ANOVA followed by Tukey’s multiple comparisons test.

### AAV9-c-fos-mediated MAPK3 knockdown inhibits ferroptosis

To determine whether AAV9-c-fos-mediated MAPK3 knockdown inhibits ferroptosis, we examined key ferroptosis-related markers by Western blotting. Compared to WT controls, APP/PS1 mice exhibited a marked pro-ferroptotic phenotype, characterized by downregulation of GPX4, its upstream regulator Nrf2, and Nrf2 target genes (HO-1, FTH1, XCT), along with upregulation of ACSL4, KEAP1, and NCOA4. Notably, AAV9-c-fos-mediated MAPK3 knockdown significantly reversed these changes, increasing the expression of GPX4, Nrf2, HO-1, FTH1, and XCT while reducing ACSL4, KEAP1, and NCOA4 levels in APP/PS1 mice (Figures 4A–H). In contrast, AAV-NC injection had no effect on these markers. Collectively, these results demonstrate that MAPK3 knockdown activates the Nrf2-GPX4 antioxidant axis and suppresses iron-dependent lipid peroxidation, thereby inhibiting ferroptosis.

**FIGURE 4:**
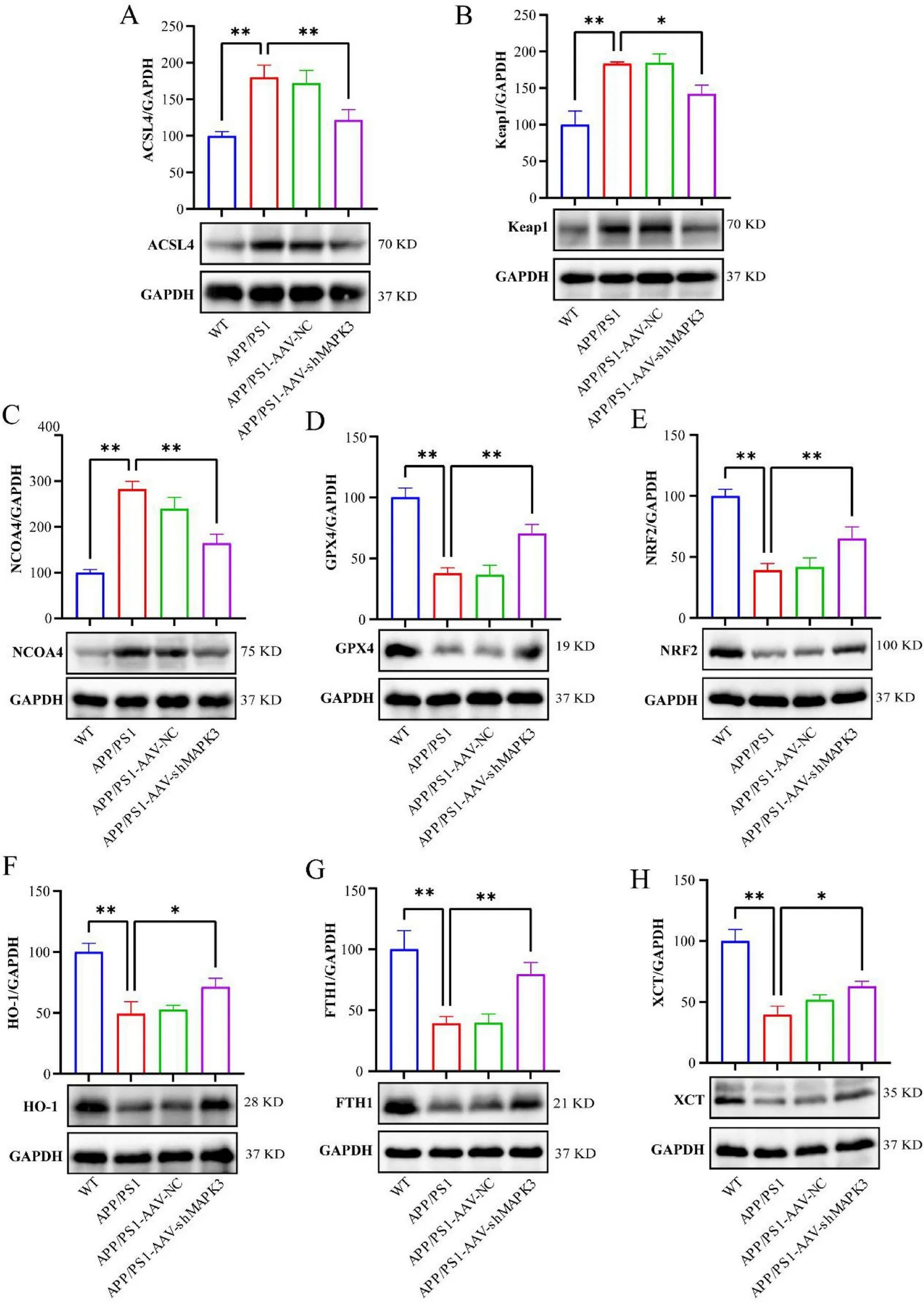
AAV9-c-fos-mediated MAPK3 knockdown inhibits ferroptosis via activating the Nrf2–GPX4 axis. **(A-H)** Representative Western blot images and corresponding quantitative analyses of ferroptosis-related markers: ACSL4 **(A)**, Keap1 **(B)**, NCOA4 **(C)**, GPX4 **(D)**, Nrf2 **(E)**, HO-1 **(F)**, FTH1 **(G)**, and XCT **(H)** in the experimental groups (*n* = 3). Data are expressed as mean ± SD, *p < 0.05, **p < 0.01, by one-way ANOVA followed by Tukey’s multiple comparisons test.

### AAV9-c-fos-mediated MAPK3 knockdown inhibits apoptosis in APP/PS1 mice

In addition to its role in regulating ferroptosis, we next investigated whether the neuroprotective effect of AAV9-c-fos-mediated MAPK3 knockdown extends to the inhibition of apoptosis, another key cell death pathway implicated in AD pathogenesis (Kumari et al., 2023). We assessed the expression of BAX and BCL2 to evaluate the apoptotic balance. APP/PS1 mice showed a significant increase in Bax and a decrease in Bcl2 compared to WT mice, indicating enhanced apoptosis (Figure 5A, B). Notably, AAV9-c-fos-mediated MAPK3 knockdown effectively reversed these changes, reducing Bax and increasing Bcl2 expression compared to the APP/PS1 group (Figures 5A, B).

**FIGURE 5:**
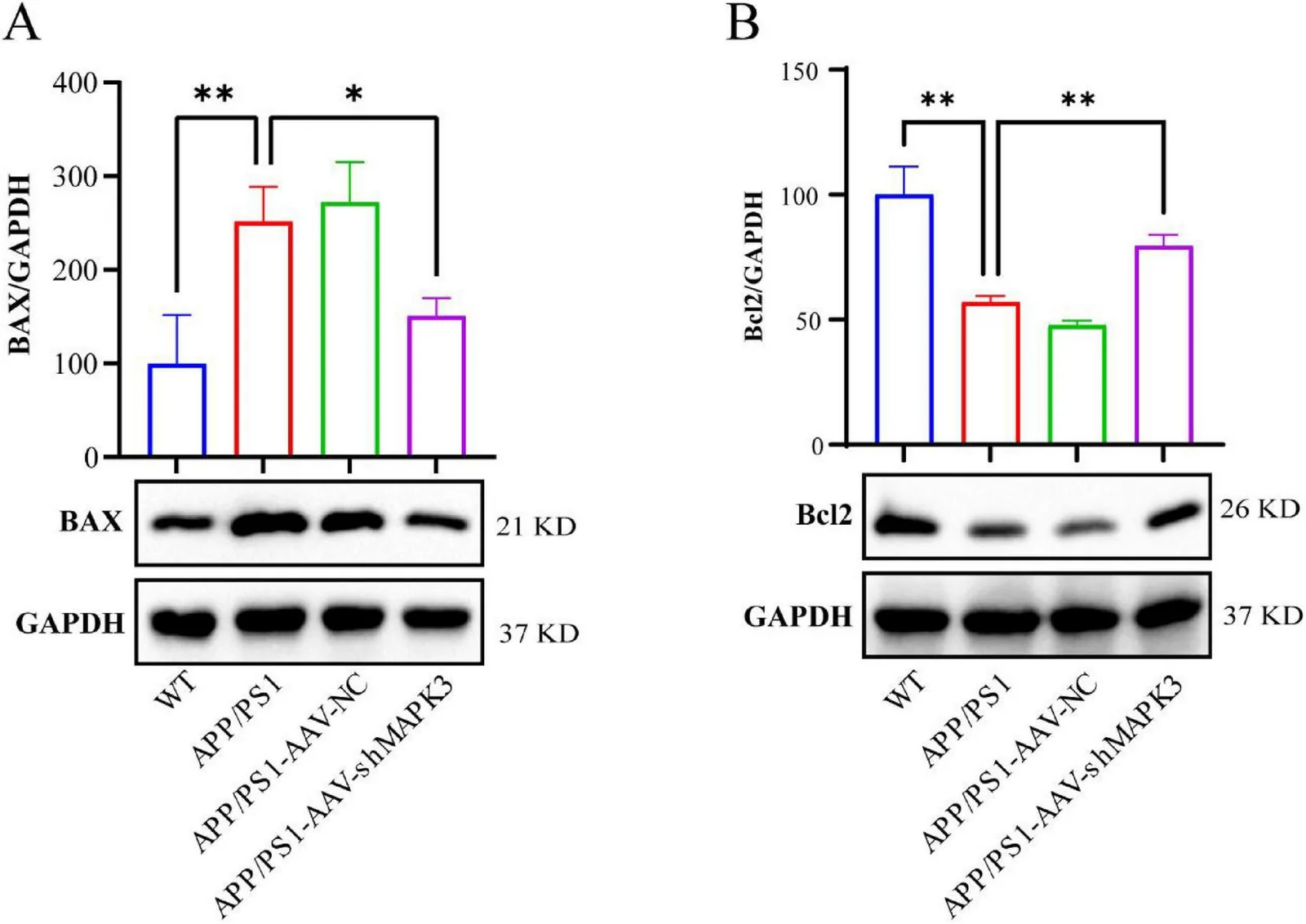
AAV9-c-fos-mediated MAPK3 knockdown attenuates apoptosis in APP/PS1 mice. **(A)** Representative Western blot images and corresponding quantitative analyses of BAX in the experimental groups (*n* = 3). **(B)** Representative Western blot images and corresponding quantitative analyses of Bcl2 in the experimental groups (*n* = 3). Data are expressed as mean ± SD, *p < 0.05, **p < 0.01, by one-way ANOVA followed by Tukey’s multiple comparisons test.

### AAV9-c-fos-mediated MAPK3 knockdown reduces markers of neuronal activity

The fluorescence intensity of c-fos was decreased in APP/PS1+AAV-shMAPK3 group compared to AAV-NC controls (Figure 6A). Meanwhile, the fluorescence intensity of ACSL4 was also decreased in APP/PS1+AAV-shMAPK3 group compared to AAV-NC controls (Figure 6B).

**FIGURE 6:**
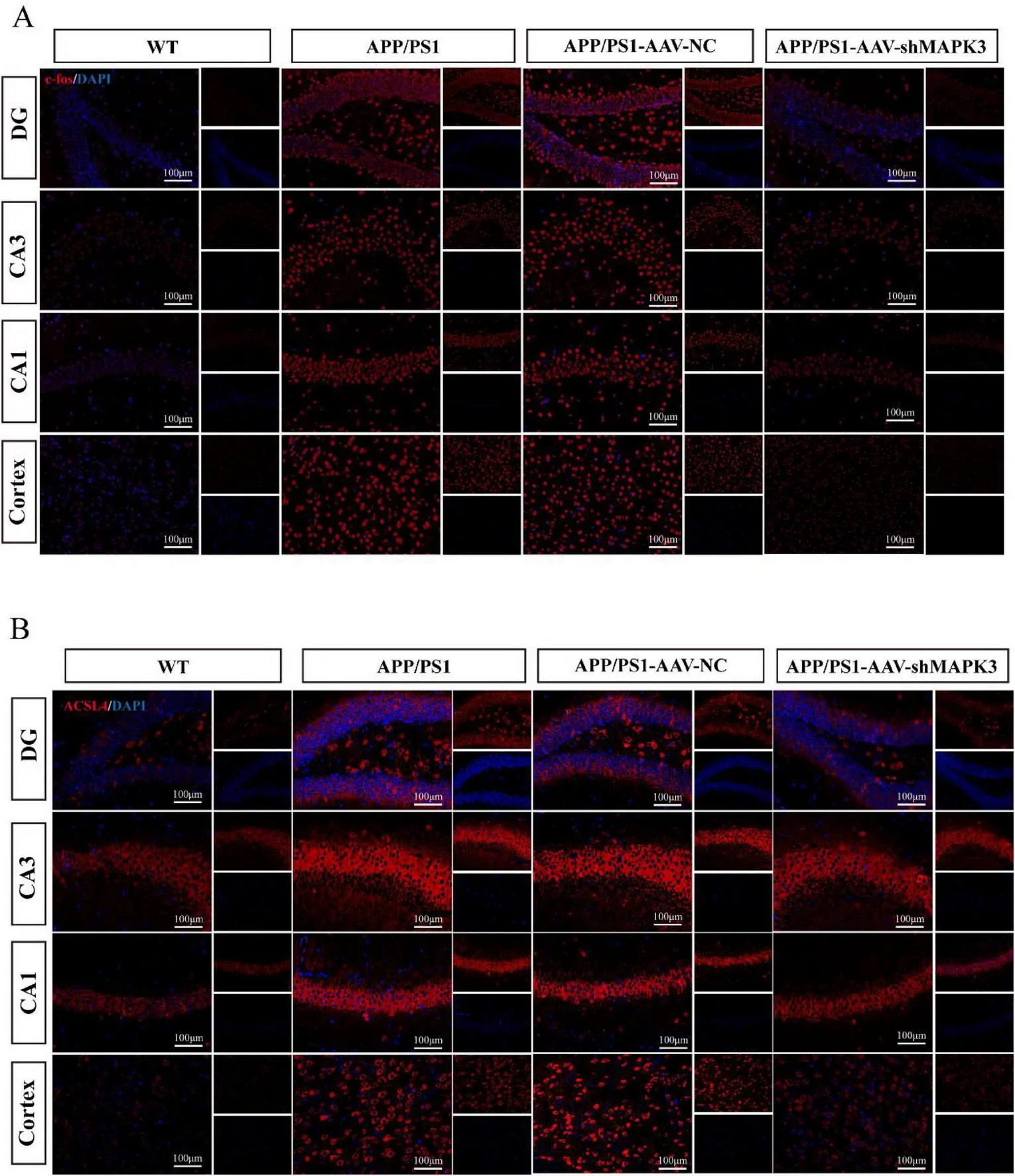
AAV9-c-fos-mediated MAPK3 knockdown reduces c-fos and ACSL4 fluorescence intensity. **(A)** Representative images of staining of c-fos in the cortex and Hippocampus (*n* = 3). **(B)** Representative images of staining of ACSL4 in the cortex and Hippocampus (*n* = 3). Representative immunofluorescence images from three independent experiments are shown. No quantitative statistical analysis was performed on these data.

### si-MAPK3 inhibits CaMKII activation in Aβ-induced HT22 cells

To further validate our *in vivo* findings, we established an *in vitro* AD model by treating HT22 hippocampal neurons with Aβ oligomers. MAPK3 knockdown was achieved using small interfering RNA (siRNA). After 48 h of transfection, cells were exposed to Aβ oligomers for an additional 24 h. Western blot analysis first confirmed efficient si-MAPK3, as evidenced by significantly reduced MAPK3 protein levels compared to the siNC group (Figure 7A). We next examined whether si-MAPK3 modulates CaMKII activation. Aβ treatment significantly increased p-CaMKII (Thr286) levels in control cells, an effect that was markedly reversed by si-MAPK3 without affecting total CaMKII expression (Figures 7B, C), confirming that MAPK3 regulates CaMKII activity in neurons.

**FIGURE 7:**
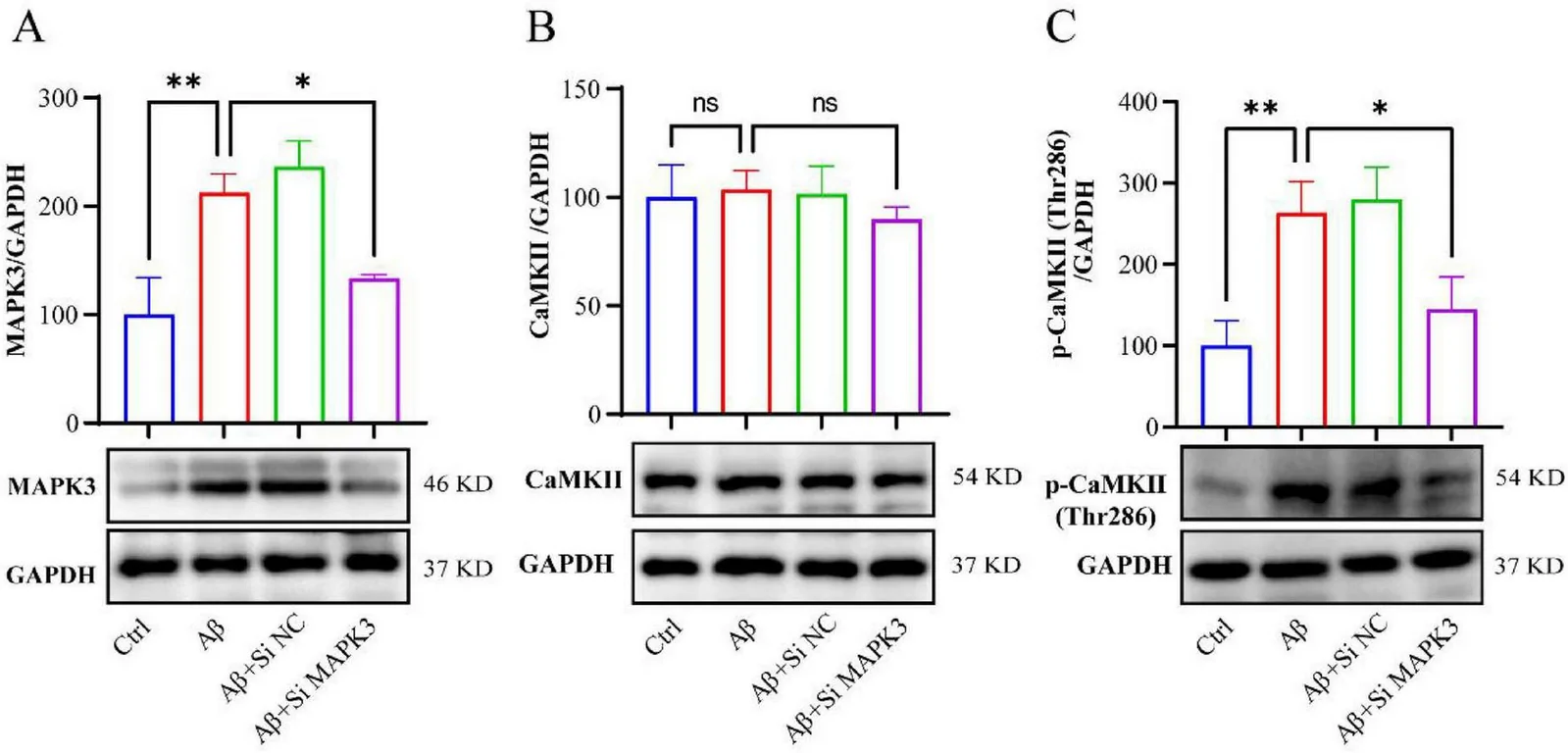
siMAPK3 suppresses CaMKII activation in Aβ-induced HT22 cells. **(A)** Representative Western blot images and quantitative analysis confirming si-MAPK3 efficiency (*n* = 3). **(B,C)** Representative Western blot images and the quantitative analysis of total CaMKII (B) and p-CaMKII (Thr286) **(C)** (*n* = 3). Data are expressed as mean ± SD, *p < 0.05, **p < 0.01, by one-way ANOVA followed by Tukey’s multiple comparisons test.

### si-MAPK3 inhibits ferroptosis in HT22 cells

To validate our *in vivo* observations, we further investigated whether si-MAPK3 inhibits ferroptosis *in vitro*. Mirroring the ferroptotic phenotype observed in APP/PS1 mice, Aβ-treated control cells displayed reduced expression of GPX4, Nrf2, HO-1, FTH1, and XCT, along with increased expression of ACSL4, KEAP1, and NCOA4 (Figures 8A–H). Notably, si-MAPK3 reversed these changes, upregulating the levels of GPX4, Nrf2, HO-1, FTH1, and XCT while reducing ACSL4, KEAP1, and NCOA4 expression (Figures 8A–H). These results recapitulate our *in vivo* findings and demonstrate that si-MAPK3 knockdown protects hippocampal neurons against Aβ-induced ferroptosis by bolstering the Nrf2-GPX4 antioxidant defense system.

**FIGURE 8:**
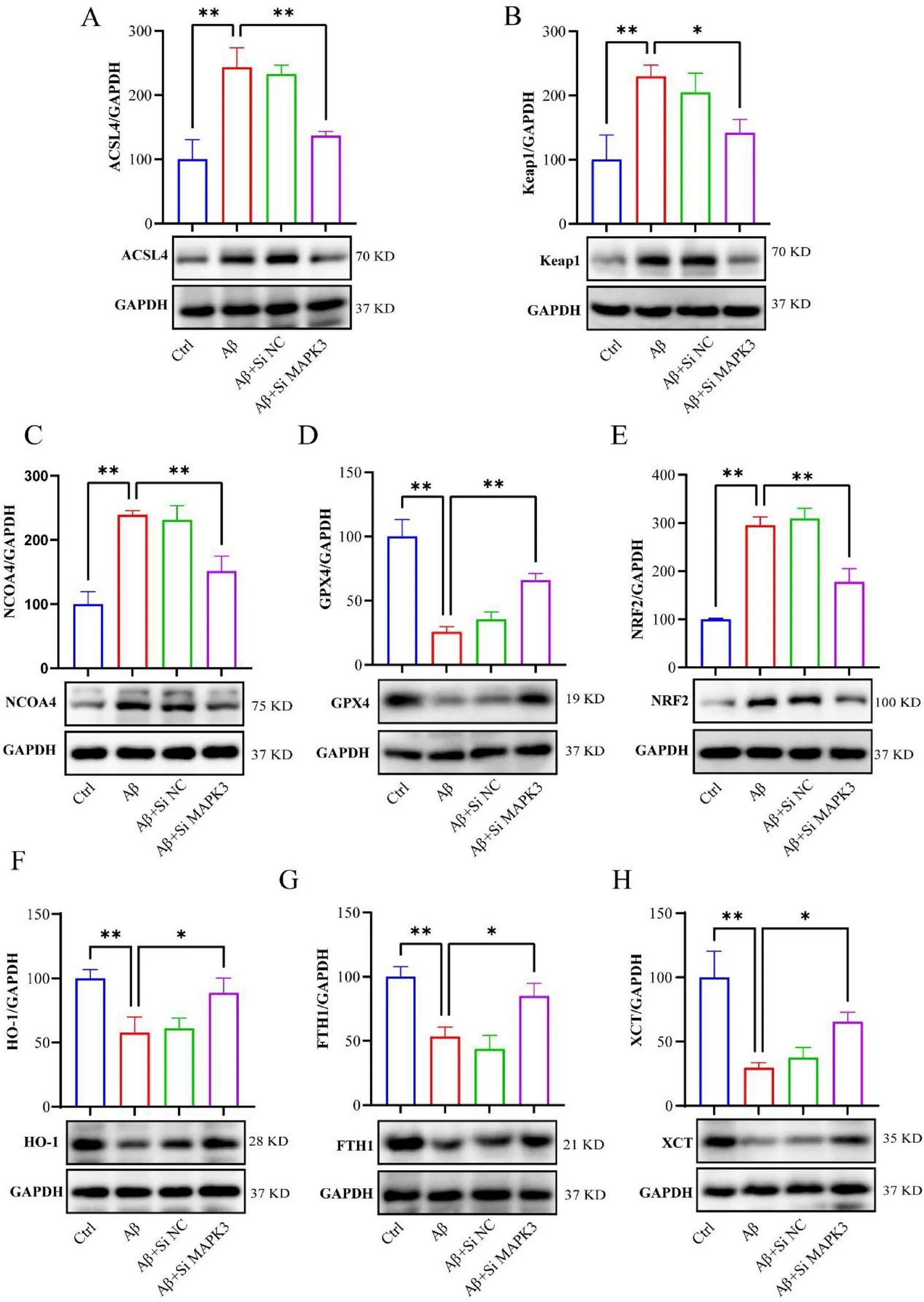
siMAPK3 Inhibits Aβ-induced ferroptosis in HT22 cells. **(A-H)** Representative Western blot images and corresponding quantitative analyses of ferroptosis-related markers: ACSL4 **(A)**, Keap1 **(B)**, NCOA4 **(C)**, GPX4 **(D)**, Nrf2 **(E)**, HO-1 **(F)**, FTH1 **(G)**, and XCT **(H)** in the HT22 cell groups (*n* = 3). Data are expressed as mean ± SD, with *p < 0.05 and **p < 0.01, by one-way ANOVA followed by Tukey’s multiple comparisons test.

### siMAPK3 inhibits apoptosis in HT22 cells

Given that MAPK3 knockdown inhibited ferroptosis, we next investigated whether it also confers protection against apoptosis. Aβ stimulation significantly increased Bax expression and decreased Bcl-2 levels in control cells, indicating a pro-apoptotic shift. This imbalance was effectively reversed by si-MAPK3, which downregulated Bax and upregulated Bcl-2 expression (Figures 9A, B). These findings demonstrate that MAPK3 knockdown inhibits neuronal apoptosis, extending its neuroprotective effects beyond ferroptosis.

**FIGURE 9:**
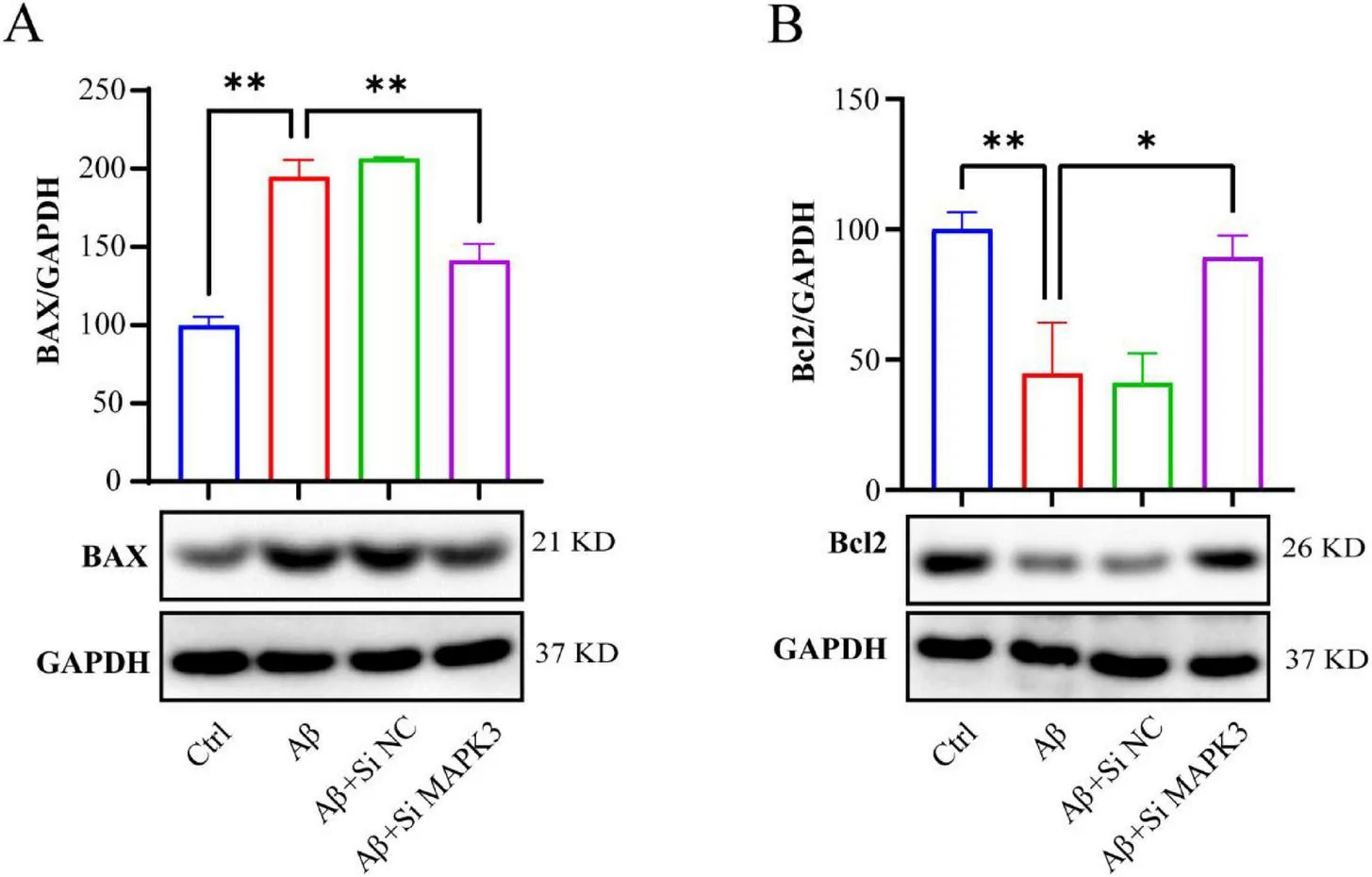
siMAPK3 inhibits Aβ-induced apoptosis in HT22 cells. **(A)** Representative Western blot images and the quantitative analysis of BAX (*n* = 3). **(B)** Representative Western blot images and the quantitative analysis of Bcl2 (*n* = 3). Data are expressed as mean ± SD, *p < 0.05, **p < 0.01, by one-way ANOVA followed by Tukey’s multiple comparisons test.

## Discussion

This research utilized a comprehensive method, ranging from MR analysis to functional validation, to address this knowledge gap. Initially, we identified ferroptosis genes with a causal link to AD and highlighted MAPK3 as a significant risk gene acting specifically through excitatory neurons. Subsequent *in vivo* and *in vitro* experiments were performed to confirm its role and clarify the underlying mechanisms. The following discussion will interpret these key findings, including the genetic causality of MAPK3, its role in mitigating cognitive deficits, and its regulation of downstream CaMKII signaling and cell death pathways such as ferroptosis and apoptosis. It is important to note that the genetic association identified by MR analysis and the mechanistic pathway elucidated through experimental validation represent distinct levels of biological inference.

Our findings provide evidence fora critical molecular pathway in which MAPK3 in excitatory neurons serves as a central node that connects genetic risk to the activation of ferroptosis and apoptosis in AD. Previous studies have implicated ERK/MAPK3 signaling in neurodegeneration (Khezri et al., 2023; Li et al., 2023); however, the specific connection to ferroptosis in excitatory neurons has not been extensively explored. The data demonstrate that the knockdown of MAPK3, both *in vivo* and *in vitro*, results in the suppression of phosphorylation of CaMKII at Thr286, a crucial event associated with aberrant neuronal excitability and synaptic dysfunction in AD. These experiments used an AAV9-c-fos approach to target neurons, but since the c-fos promoter is activity-dependent and MAPK3 is widely expressed, the MAPK3-CaMKII axis might indicate a general neuronal stress response, not just an excitatory neuron-specific pathway. This inhibition of CaMKII activation appears to be a pivotal upstream event, given that hyperactive CaMKII signaling is recognized as a contributor to excitotoxicity and calcium overload, which are characteristic features of AD pathology (Ghosh and Giese, 2015; Tang et al., 2025). Furthermore, our data are consistent with a mechanistic link between MAPK3 signaling and the suppression of the Nrf2-GPX4 antioxidant axis, although the intervening molecular steps remain to be defined.

The knockdown of MAPK3 significantly upregulated Nrf2 and its downstream targets, including GPX4, heme oxygenase-1 (HO-1), ferritin heavy chain 1 (FTH1), and the cystine/glutamate antiporter (xCT), while concurrently downregulating negative regulators such as Kelch-like ECH-associated protein 1 (KEAP1) and pro-ferroptotic mediators like acyl-CoA synthetase long-chain family member 4 (ACSL4) and nuclear receptor coactivator 4 (NCOA4). This coordinated regulatory mechanism effectively enhanced cellular defenses against iron-dependent lipid peroxidation, the fundamental biochemical process underlying ferroptosis. The Nrf2-GPX4 axis is a well-established master regulatory pathway that mitigates ferroptosis in various disease contexts (Lv et al., 2024; Wen et al., 2023; Yang et al., 2023). Our findings suggest MAPK3 as a previously underappreciated upstream regulator of this pathway, specifically in AD-vulnerable excitatory neurons. The convergence of MAPK3 on both CaMKII (excitability) and the Nrf2-GPX4 axis (antioxidant defense) may offer a unified mechanism that helps to explain how genetic susceptibility may lead to increased neuronal vulnerability to multiple cell death pathways.

Inhibition of MAPK3 exerts extensive neuroprotective effects by simultaneously suppressing ferroptosis and apoptosis. This inhibition alters the Bax/Bcl-2 ratio toward an anti-apoptotic state, thereby implicating mitochondrial and caspase pathways. Such dual functionality is particularly relevant in AD, where multiple forms of cell death occur concurrently (Chan et al., 2022). MAPK3 may modulate shared intermediates, such as reactive oxygen species (ROS) and mitochondrial dysfunction, across these pathways. For instance, re-establishing the Nrf2-GPX4 axis mitigates oxidative stress, which also precipitates apoptosis. Consequently, targeting MAPK3 could prove more efficacious than focusing on individual pathways like GPX4 or caspases, given the convergent nature of neurodegeneration. The specificity for excitatory neurons is particularly critical, as these neurons are highly susceptible in AD, and their hyperexcitability contributes to cognitive decline (Xu et al., 2023; Zhang et al., 2025). Demonstrating cognitive improvement in APP/PS1 mice through targeted intervention in these neurons underscores the potential for functional recovery by rectifying death signaling in this specific cell population, highlighting the importance of cell-type-specific therapeutic strategies.

From an immunological and broader pathophysiological standpoint, although our study did not specifically address neuroinflammation, the elucidated MAPK3-Nrf2 axis holds significant implications for the brain’s immune microenvironment. Nrf2 serves as a pivotal regulator of antioxidant and anti-inflammatory pathways, playing a critical role in maintaining cellular redox homeostasis and modulating inflammatory responses (Saha et al., 2020; Staurengo-Ferrari et al., 2019; Wang et al., 2024). Its activation through MAPK3 knockdown is likely to confer protective effects not only on neurons but also on glial cells, potentially mitigating pro-inflammatory activation of microglia and astrocytes induced by oxidative stress and neuronal damage. This is particularly pertinent given that neuroinflammation is a critical factor in the progression of AD (Parhizkar and Holtzman, 2022). Additionally, the mitogen-activated protein kinase (MAPK) family, which encompasses MAPK3 (ERK1), plays a crucial role in immune signaling and stress response mechanisms (Vervaeke and Lamkanfi, 2025; Zhang and Zhang, 2022). Dysregulation of MAPK pathways, including p38 MAPK, has been associated with AD-related neuroinflammation and tau pathology (Detka et al., 2024; Perea et al., 2022). The possibility that MAPK3 may also influence inflammatory processes through these interconnected pathways warrants further investigation.

Our findings indicate that the knockdown of MAPK3 leads to the inhibition of aberrant neuronal activity, as evidenced by reduced c-fos expression, and a decrease in cell death. This reduction may indirectly mitigate the chronic inflammatory stimuli that compromised neurons impart to the adjacent glial cells. Consequently, targeting neuronal MAPK3 may offer a dual therapeutic advantage: direct neuroprotection through the inhibition of ferroptosis and apoptosis, and indirect modulation of the neuroimmune environment by enhancing Nrf2-mediated anti-inflammatory signaling pathways. This positions MAPK3 not solely as a regulator of cell death but also as a modulator of neuron-glia interactions that perpetuate disease pathology. Thus, MAPK3 may represent a multifaceted target for therapeutic intervention, aligning with the intricate and multifactorial nature of AD.

When interpreting our findings, it’s important to note several limitations. MAPK3 (ERK1) is a multifunctional kinase with various targets, and while our data suggest its role in the CaMKII-Nrf2-GPX4 axis and ferroptosis, it may also influence AD through other mechanisms. For instance, ERK signaling has been implicated in neuroinflammation, tau hyperphosphorylation (a hallmark of AD), and the regulation of synaptic plasticity and function (Chen et al., 2021; Indrigo et al., 2023; Zhang et al., 2025). Additionally, MAPK3 may interact with other members of the MAPK family to modulate neuronal stress responses (Gupta et al., 2025; Numakawa and Kajihara, 2026). Therefore, although we propose a pathway connecting MAPK3 to ferroptosis in excitatory neurons, MAPK3 likely affects multiple pathways, with their impact varying by disease context. Second, although MR supported a causal genetic association between MAPK3 and AD risk, the exact molecular intermediates connecting MAPK3 signaling to the Nrf2-GPX4 antioxidant system and to apoptosis remain unclear. Moreover, because the study did not include rescue experiments (e.g., restoring CaMKII activity in MAPK3-knockdown cells) or pharmacological validation of the proposed MAPK3-CaMKII signaling cascade, the mechanistic conclusions should be interpreted as supporting a plausible mechanism rather than definitively establishing this signaling pathway. Third, our experiments mainly used APP/PS1 transgenic mice and Aβ-treated HT22 cells, focusing on amyloid pathology, so further research is needed to see if these mechanisms apply to tau-driven neurodegeneration or other AD subtypes. Fourth, while MR provided strong genetic evidence, confirming a direct link between MAPK3 activity and ferroptosis markers in human AD brains is crucial for clinical relevance, and the therapeutic potential of MAPK3 inhibition needs exploration. Future studies should use conditional knockouts, combinatorial methods, and human tissue validation to clarify these pathways’ roles and move toward clinical applications.

In conclusion, this study synthesizes genetic epidemiology with functional neuroscience to provide evidence for the role of MAPK3 in excitatory neurons as a critical factor linking genetic risk to ferroptosis and the pathogenesis of AD. Whereas MR supports a causal genetic association, our experimental findings demonstrate that the knockdown of MAPK3 ameliorates cognitive deficits in AD mouse models and is associated with attenuated CaMKII activation together with the inhibition of both ferroptotic and apoptotic cell death through restoration of the Nrf2-GPX4 antioxidant axis. These results collectively support a plausible mechanistic pathway, although rescue experiments and pharmacological validation are required to establish the MAPK3-CaMKII signaling cascade definitively. These results help to bridge the divide between population-level genetic risk and cellular vulnerability, and support MAPK3 as a central regulatory node in multiple neurodegenerative pathways. Therefore, targeting MAPK3 or its downstream may represent a promising multi-modal therapeutic approach for AD, offering a refined framework for the development of interventions that specifically address neuronal susceptibilities associated with iron-dependent cell death.

## Data Availability

The original contributions presented in this study are included in this article/Supplementary material, further inquiries can be directed to the corresponding authors.

## Footnotes

1
http://www.zhounan.org/ferrdb/current/

2
https://www.eqtlgen.org/cis-eqtls.html

3
https://storage.googleapis.com/finngen-public-data-r12/summary_stats/release/finngen_R12_G6_ALZHEIMER.gz

4
https://www.gtexportal.org/home/

5
http://bioinfo.szbl.ac.cn/scQTLbase/Home/
