# Supplementary material for: MAPK3 as a ferroptosis driver in excitatory neurons in Alzheimer’s disease: from genetic causal inference to in vivo functional validation
Source: Front Aging Neurosci. 2026 Sep 24;18:1868908. doi: 10.3389/fnagi.2026.1868908 (PMC13645776; doi:10.3389/fnagi.2026.1868908)

Figure 3. (A) MAPK3   and GAPDH

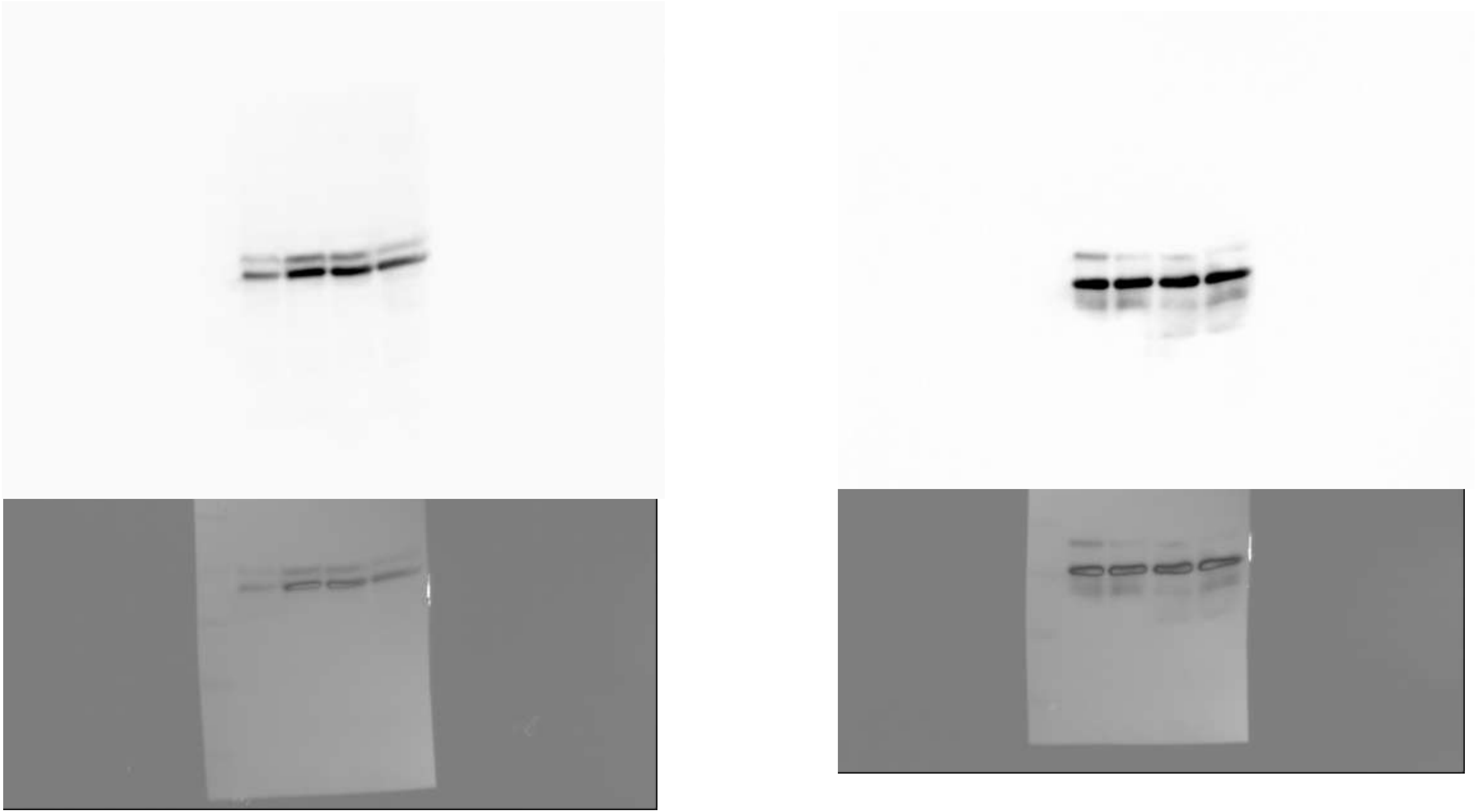

Figure 3. (B) CaMKII   and GAPDH

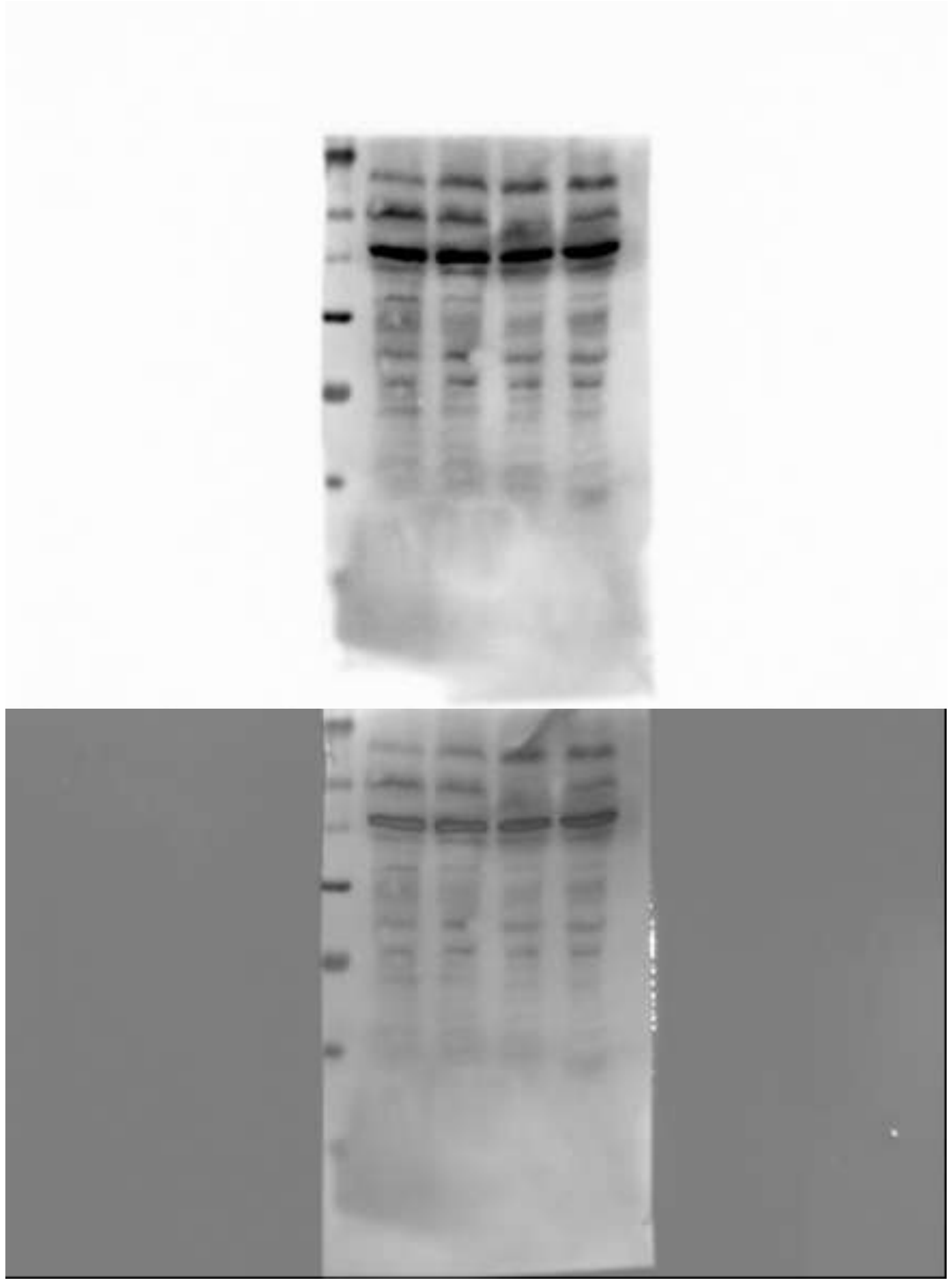

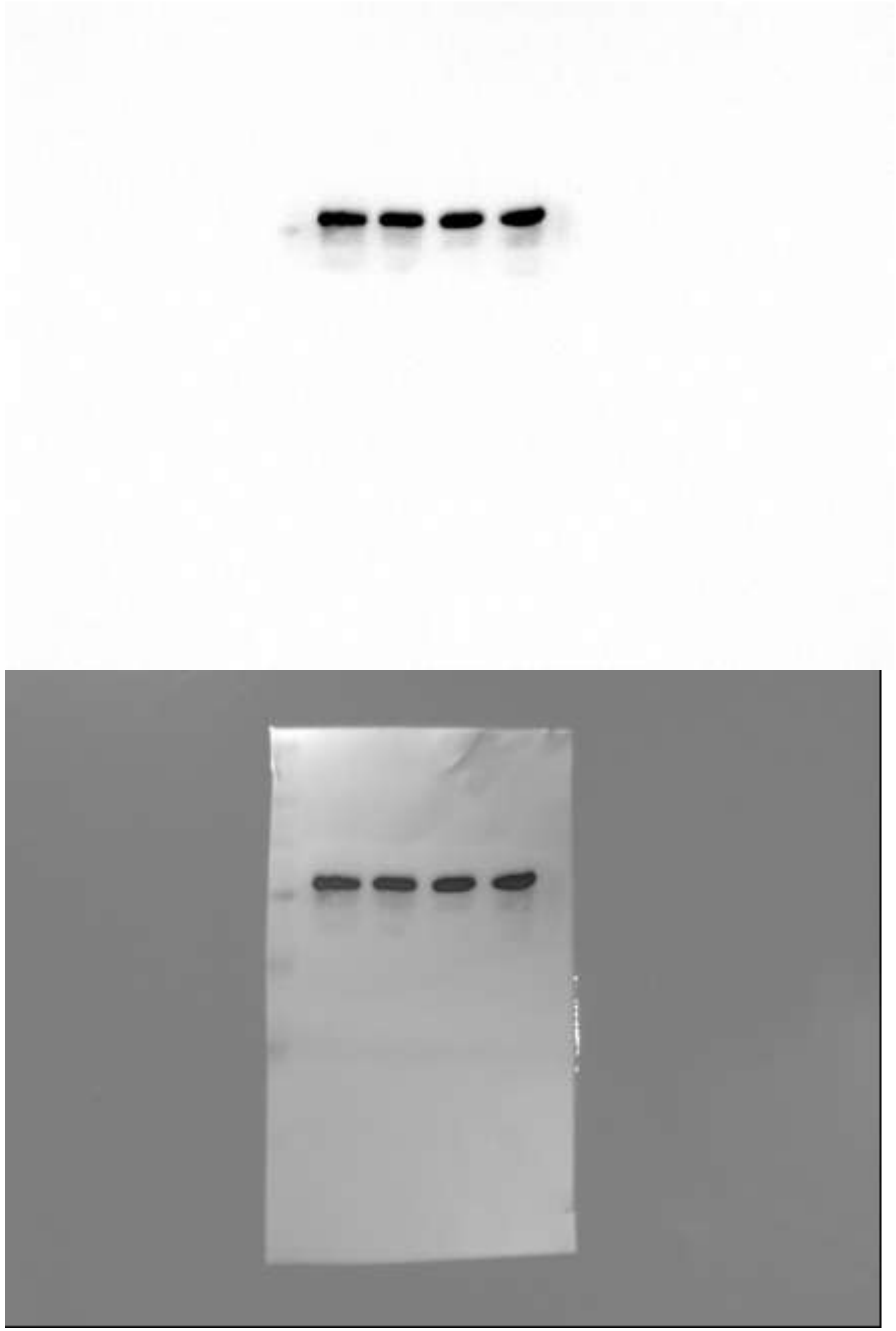

Figure 3. (C) p-CaMKII   and GAPDH

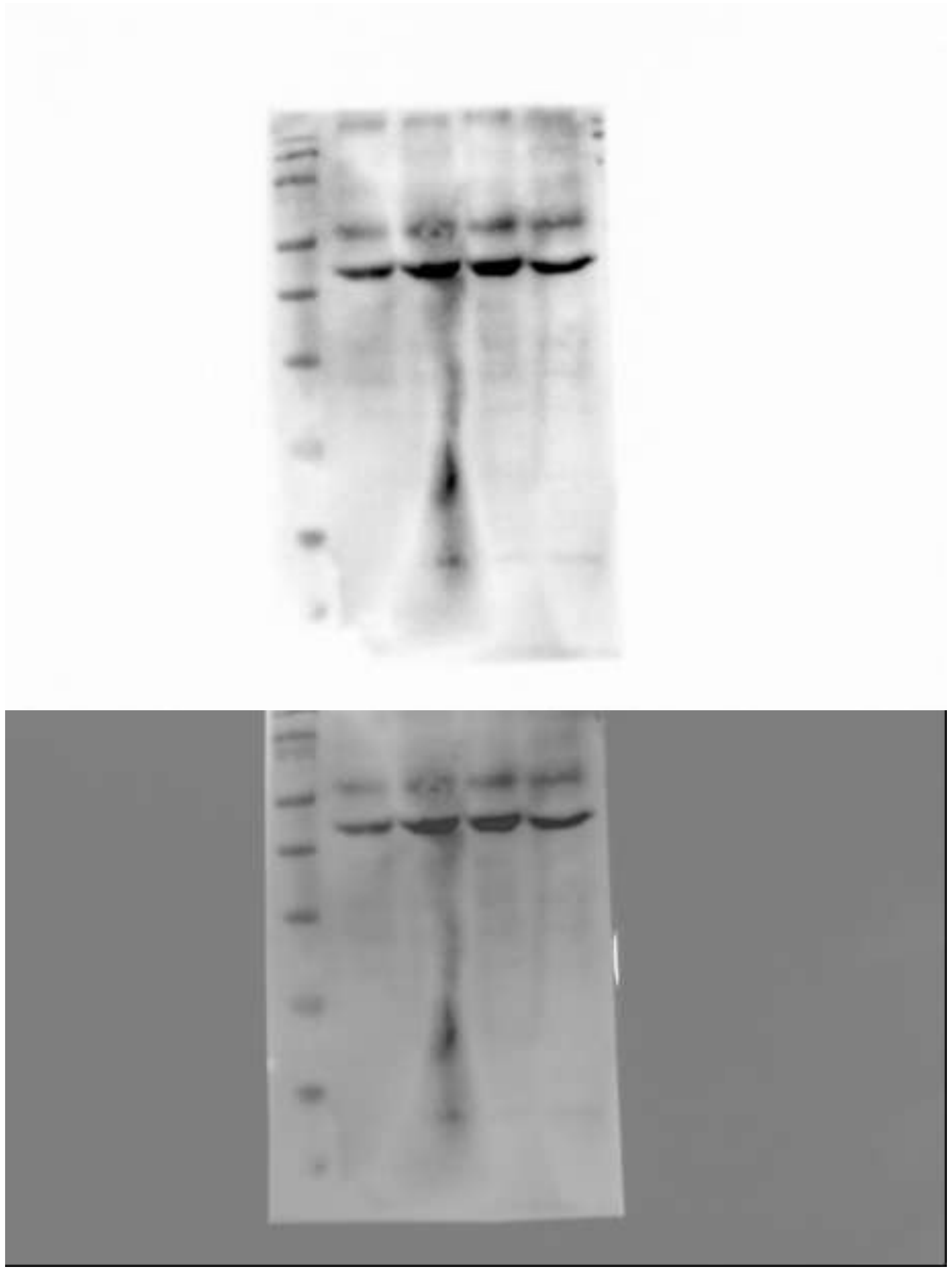

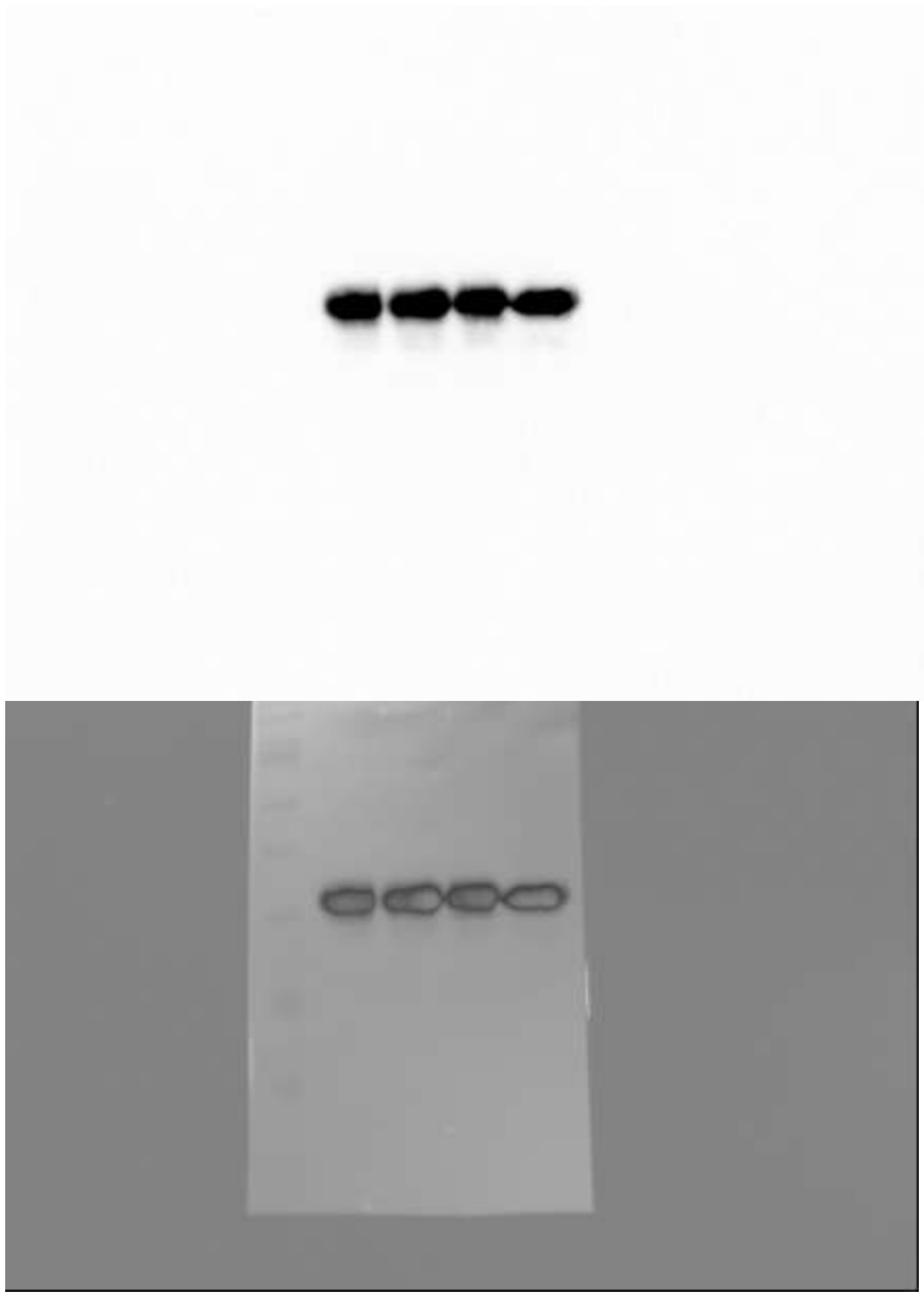

Figure 4. (A) ACSL4 and GAPDH

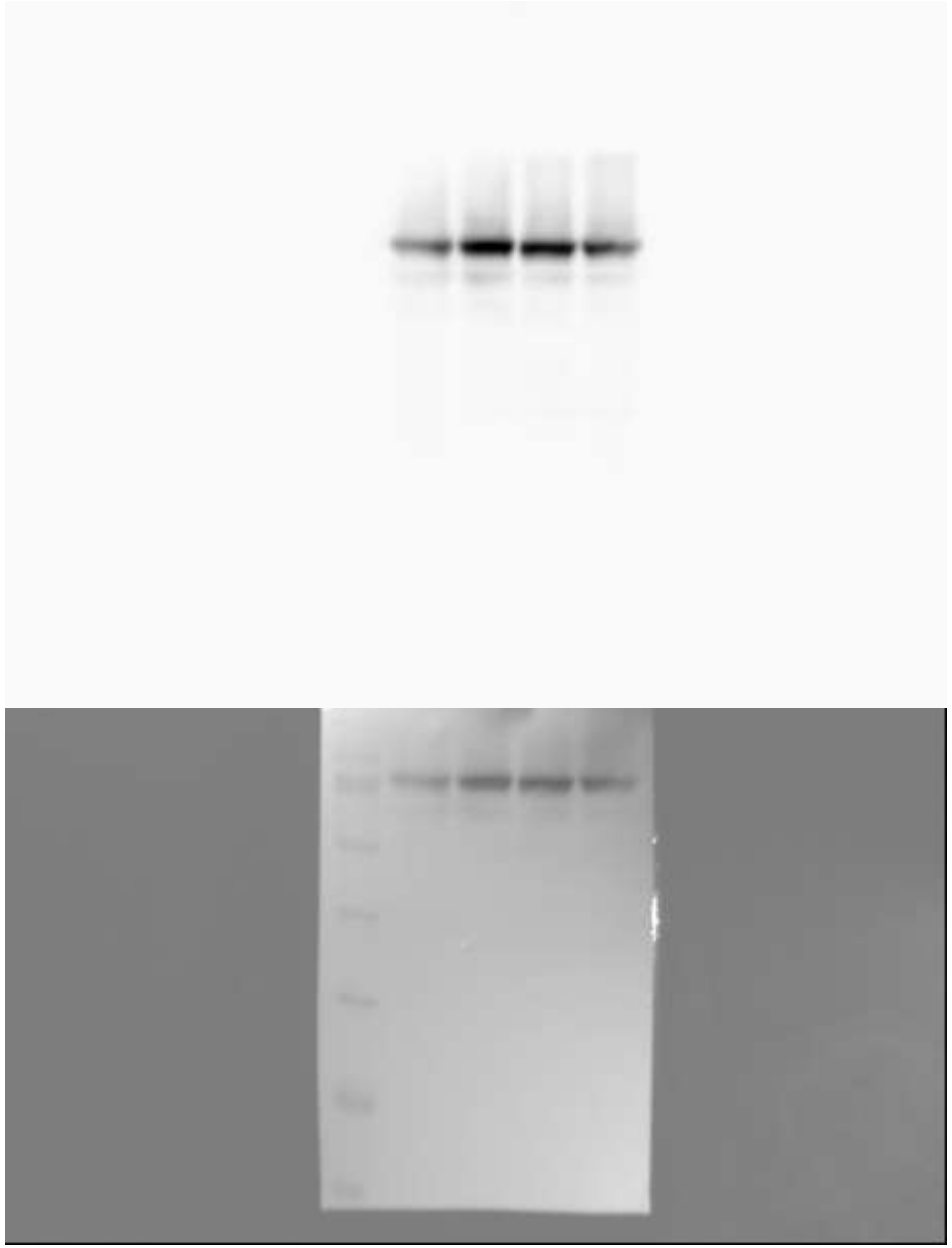

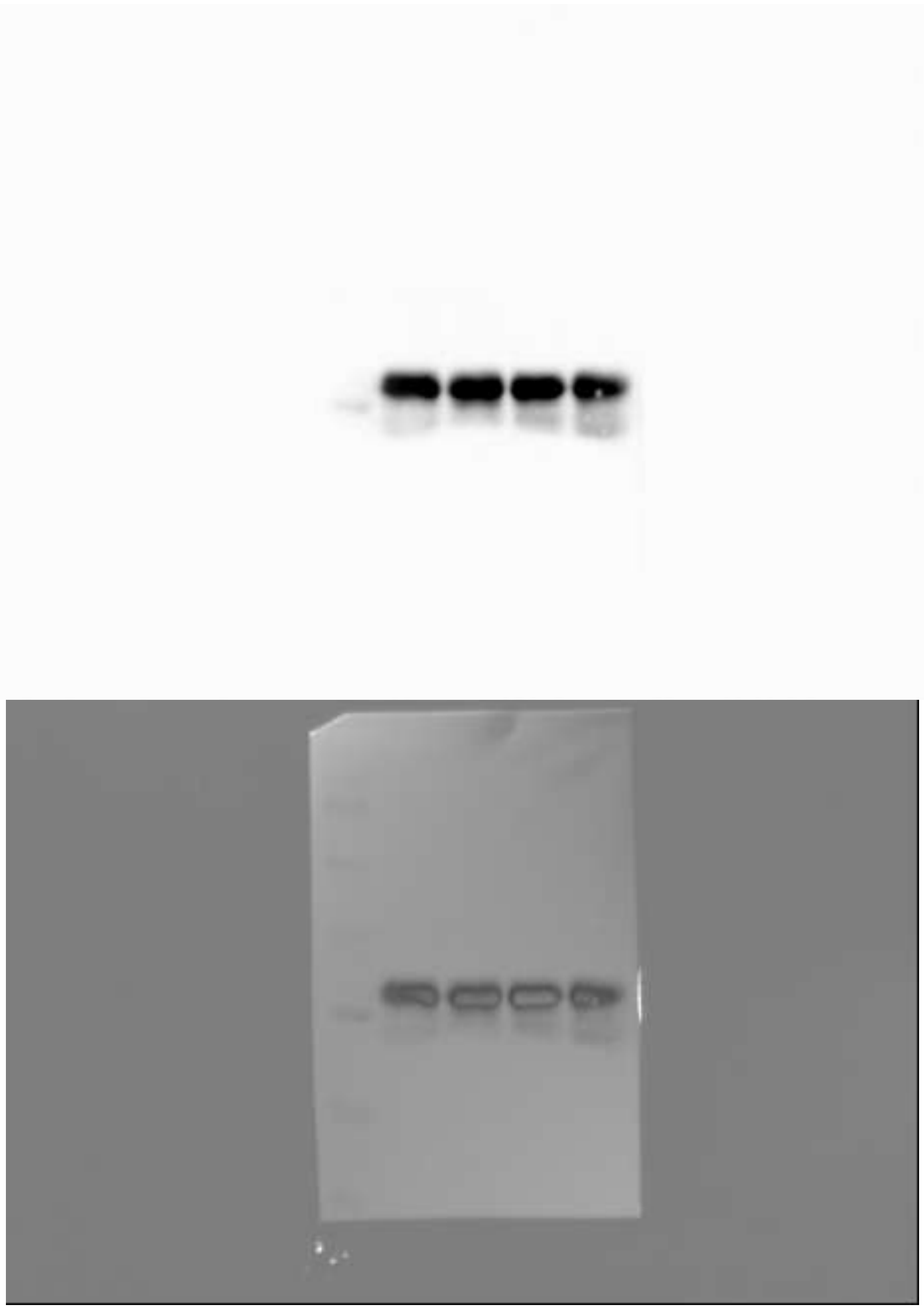

Figure 4. (B) Keap1 and GAPDH

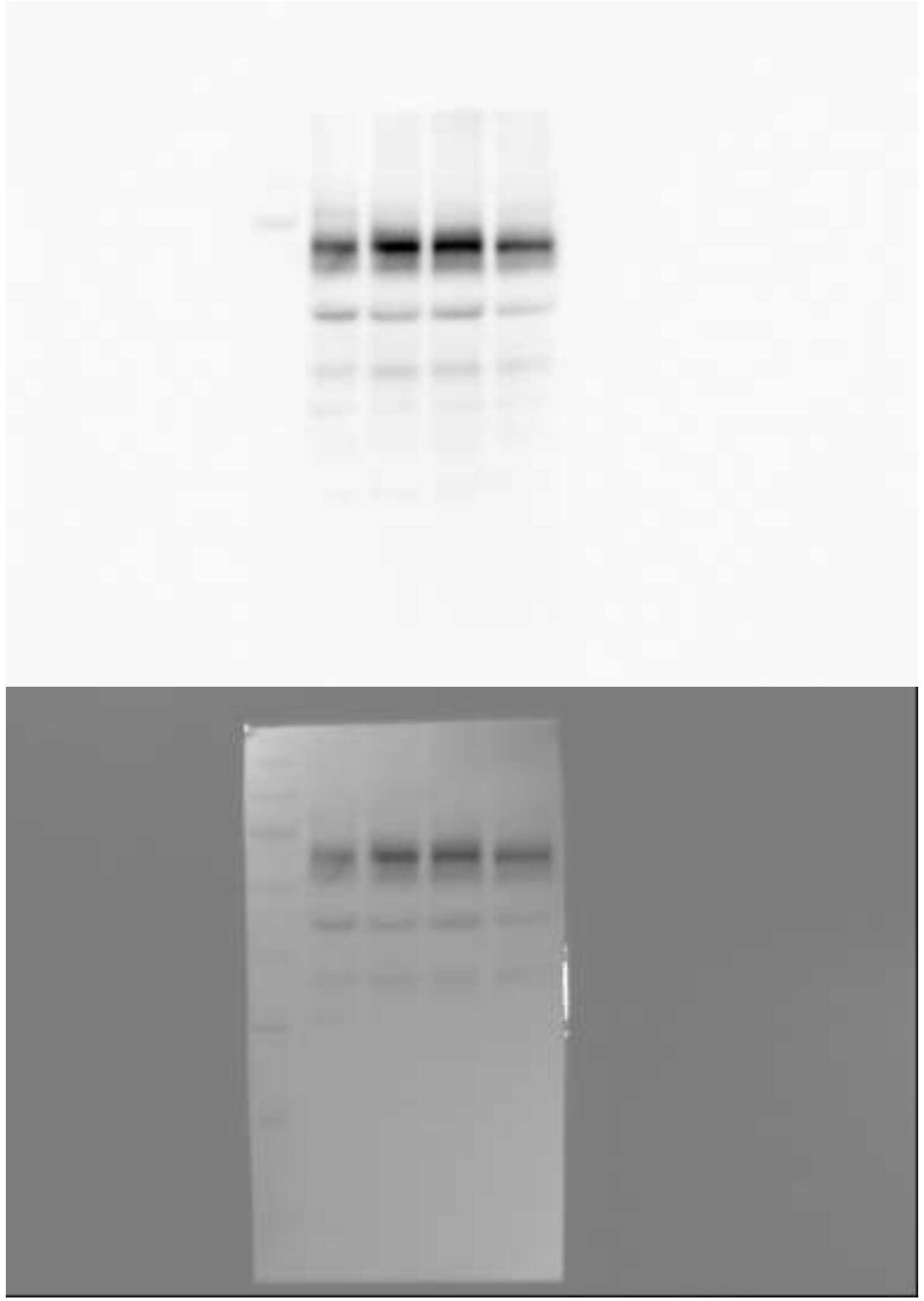

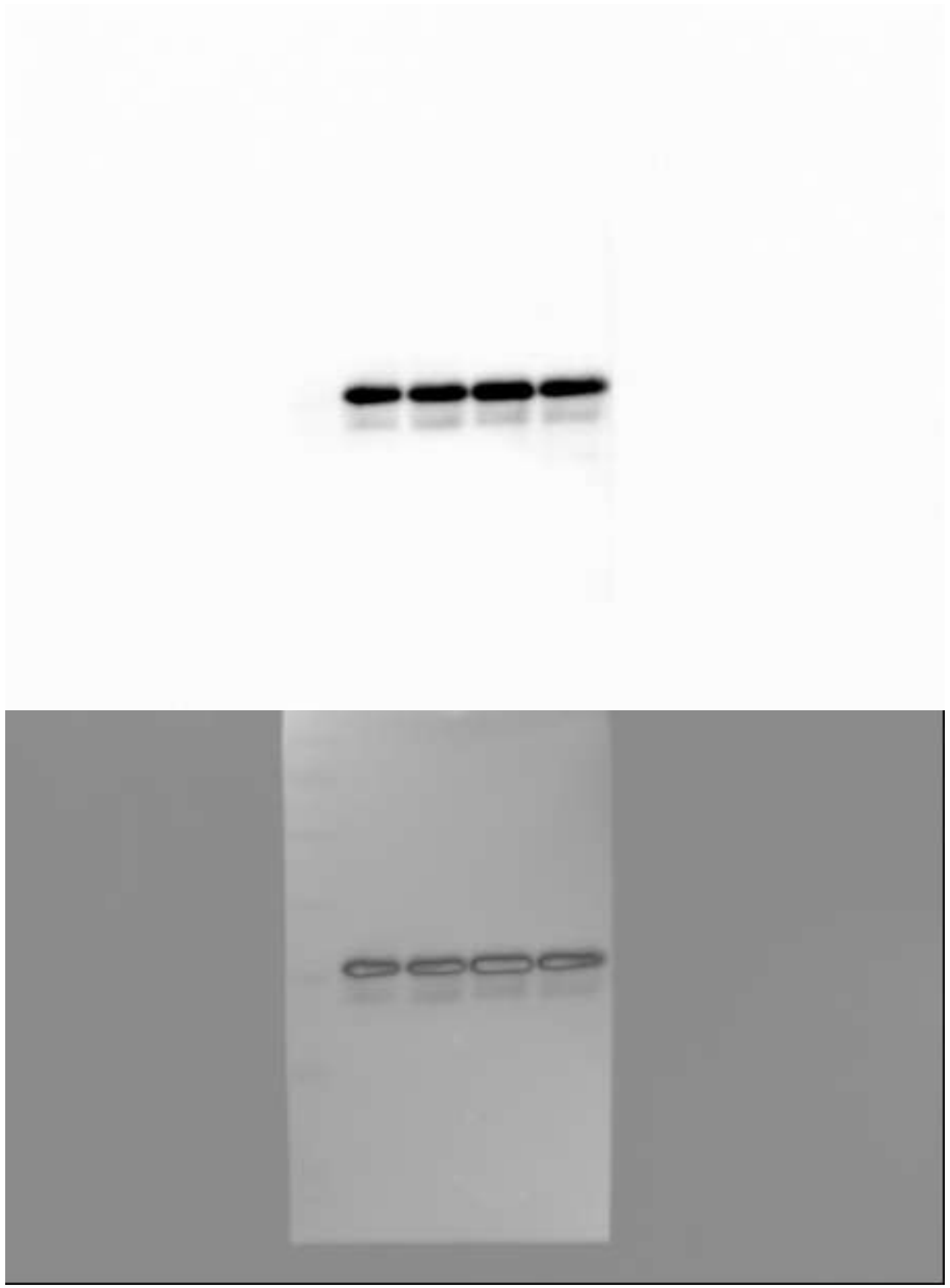

Figure 4. (C) NCOA4 and GAPDH

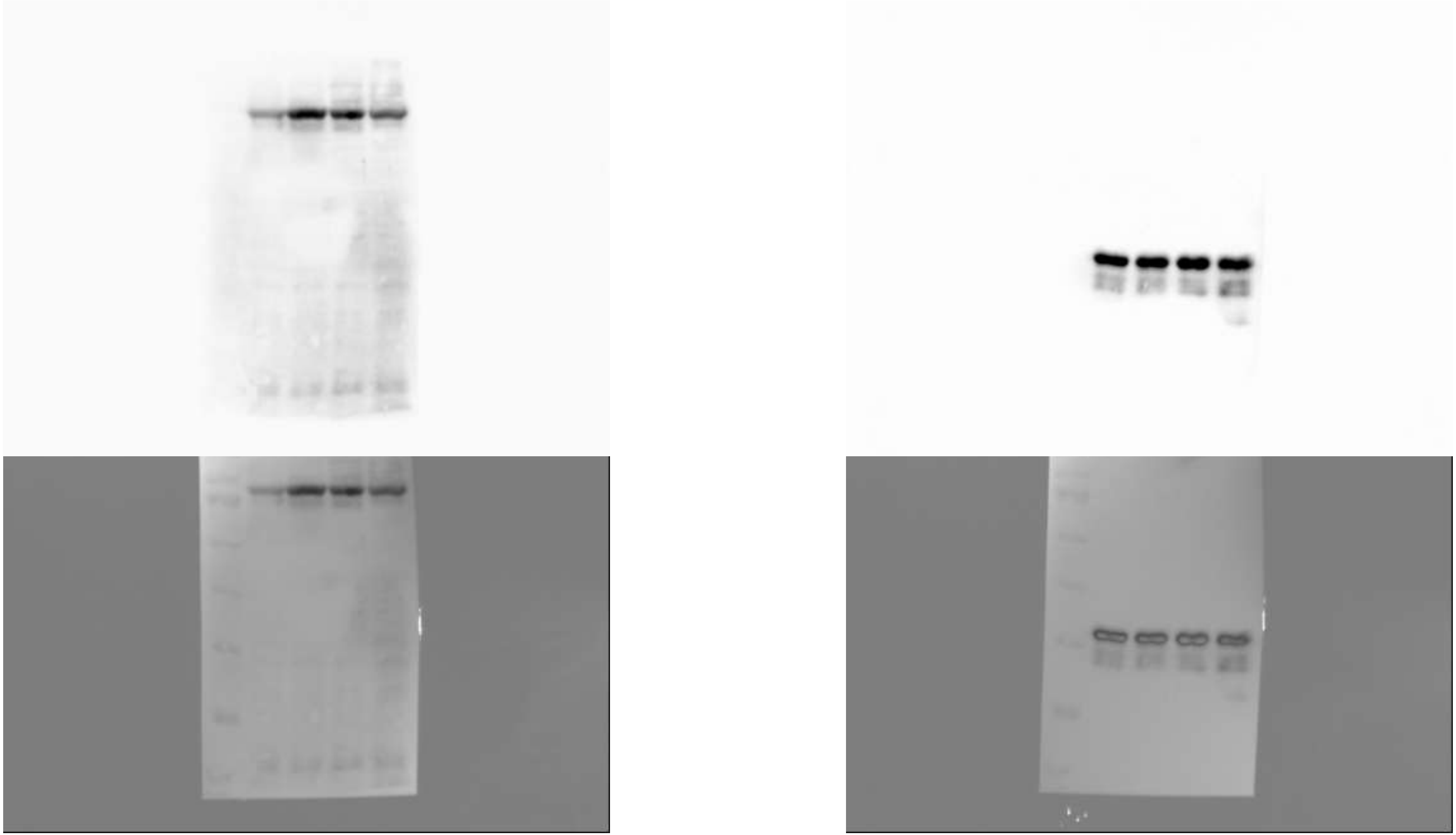

Figure 4. (D) GPX4 and GAPDH

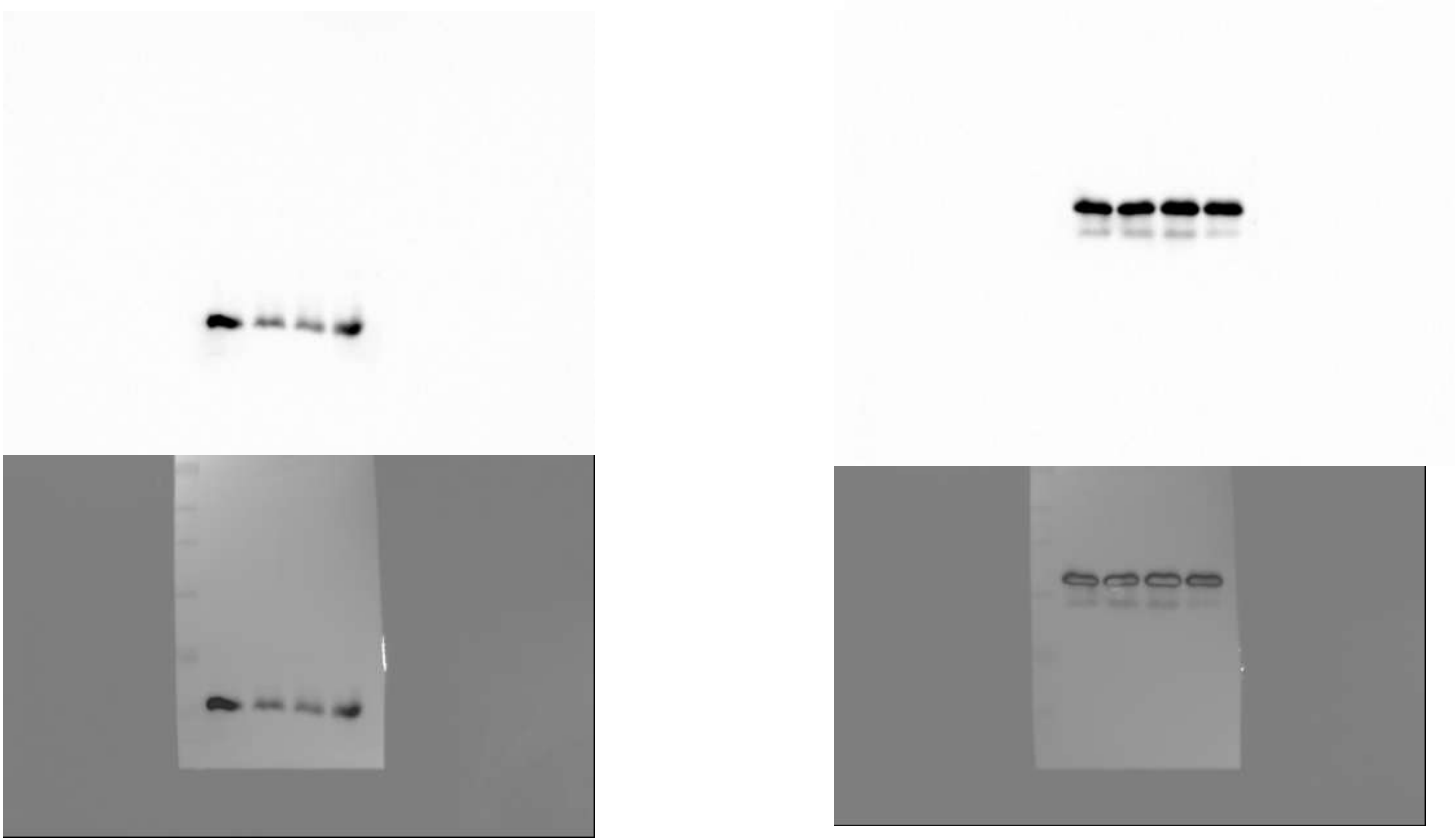

Figure 4. (E) NRF2 and GAPDH

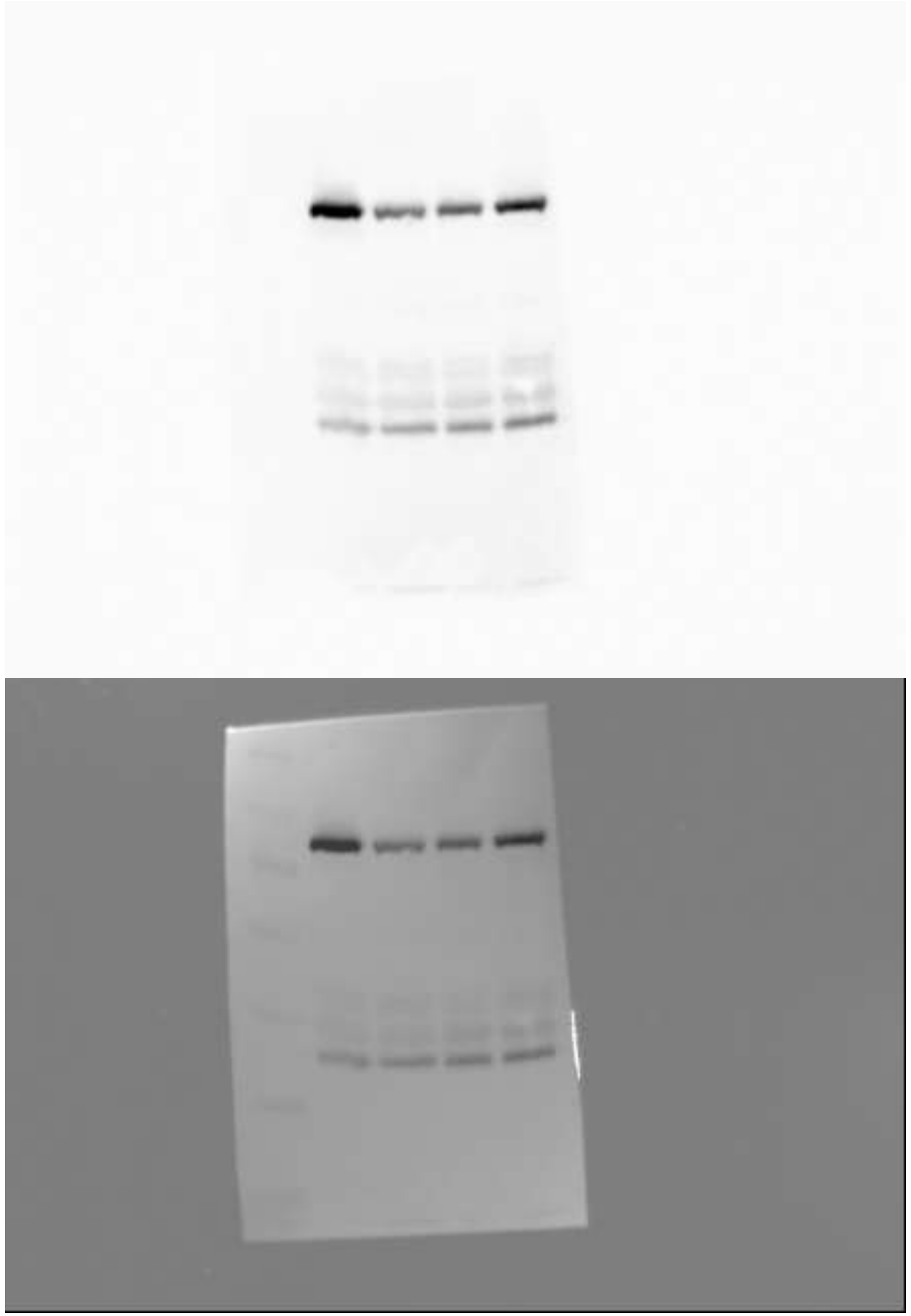

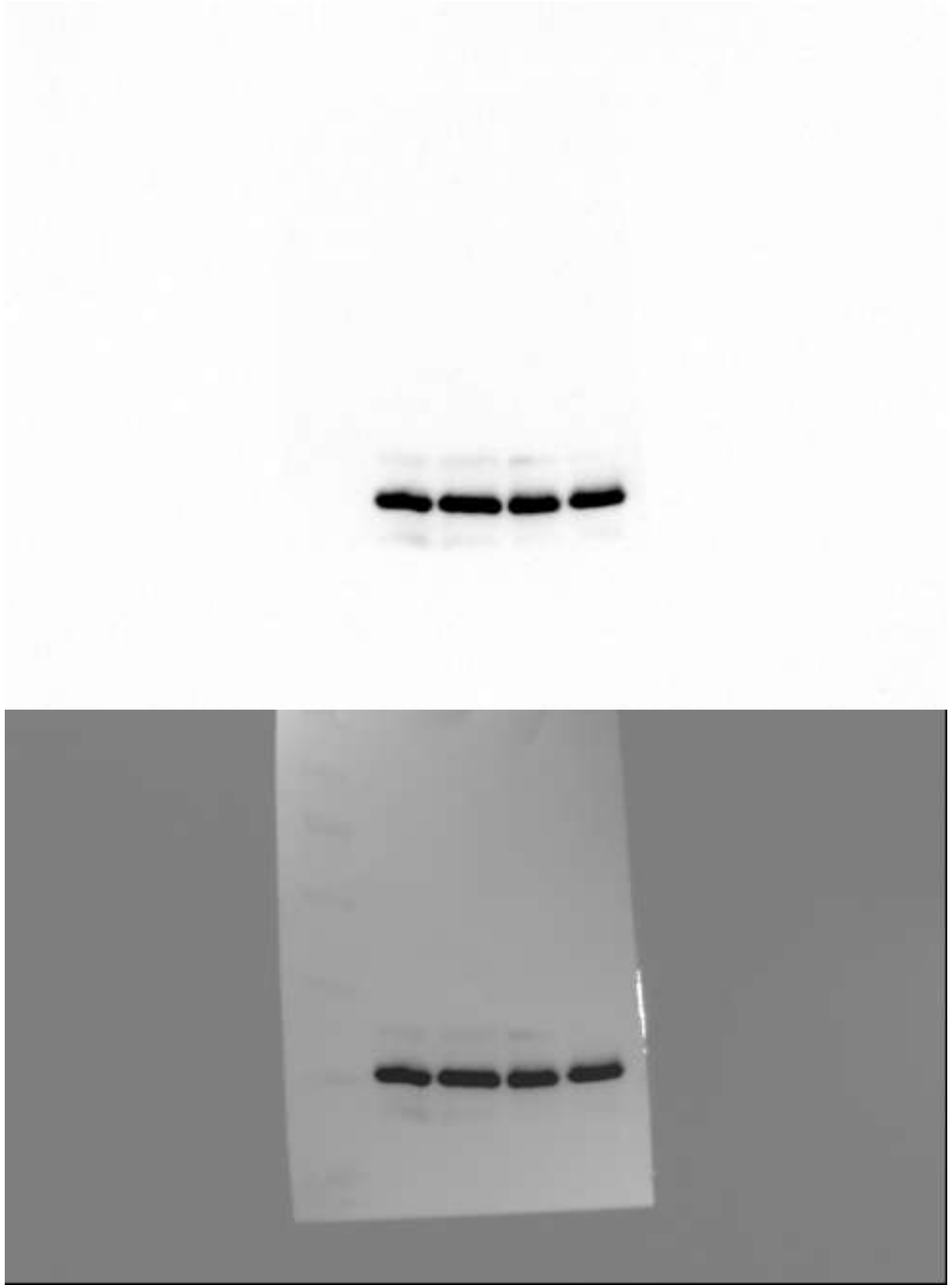

Figure 4. (F) HO-1 and GAPDH

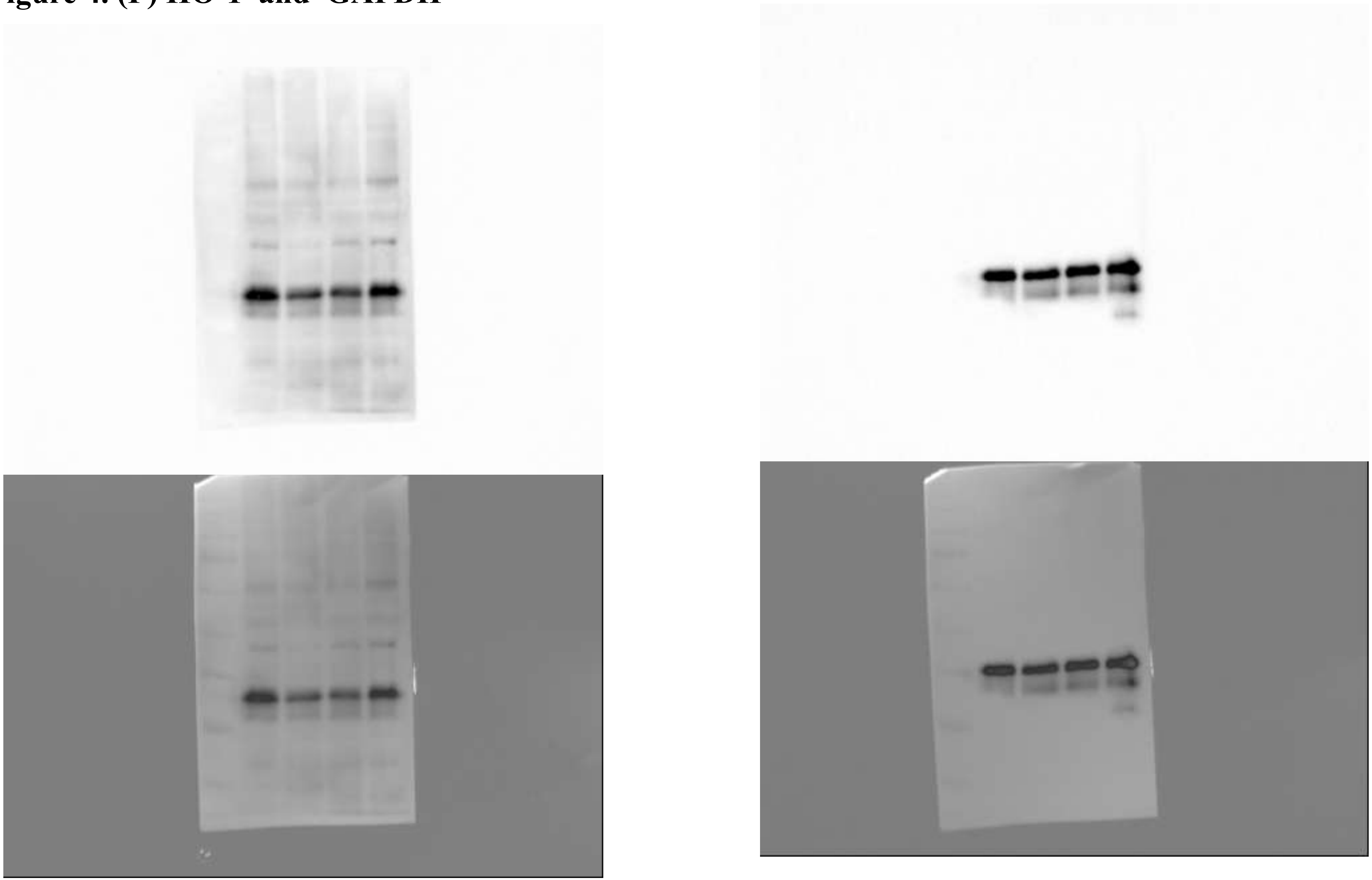

Figure 4. (G) FTH1 and GAPDH

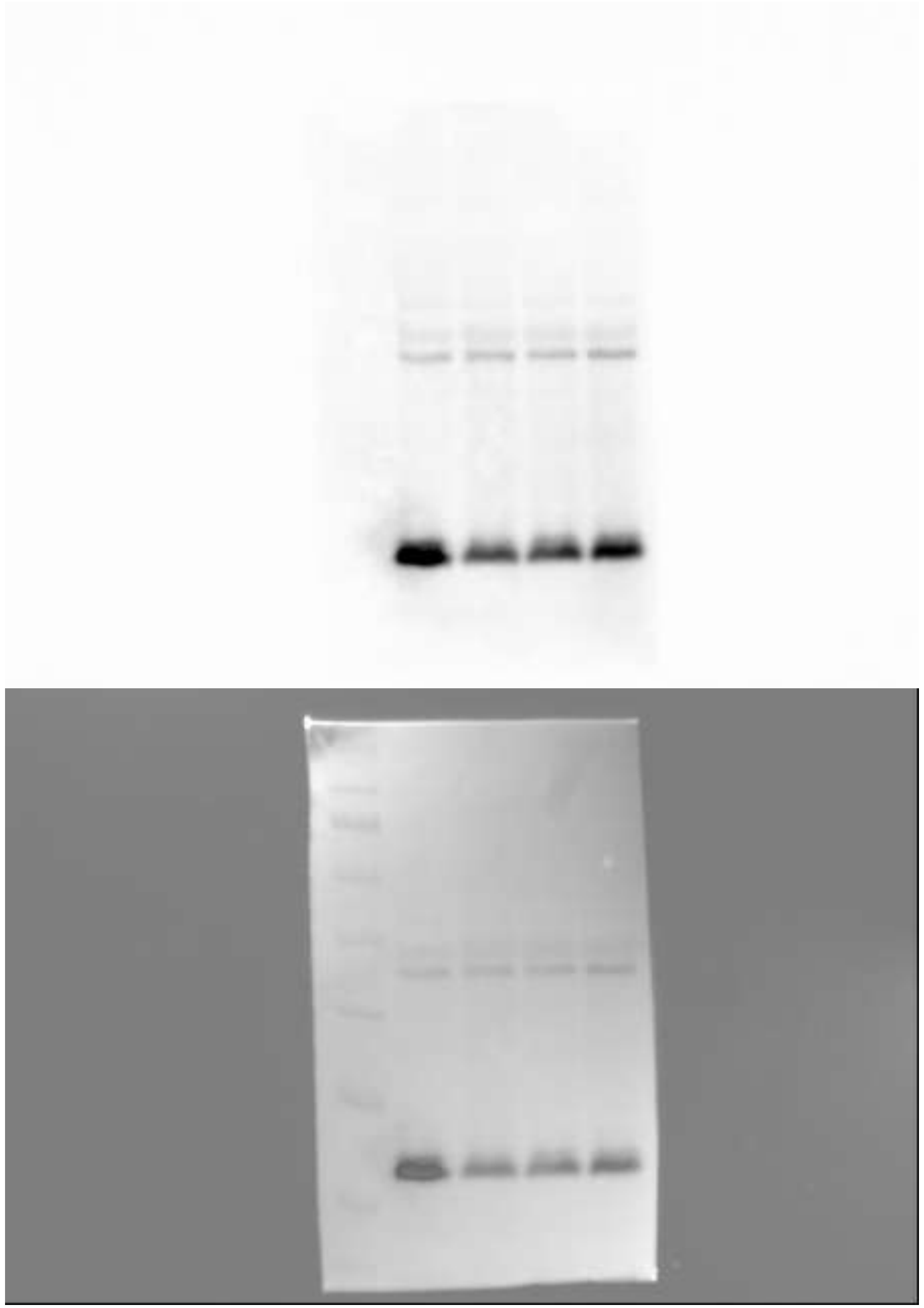

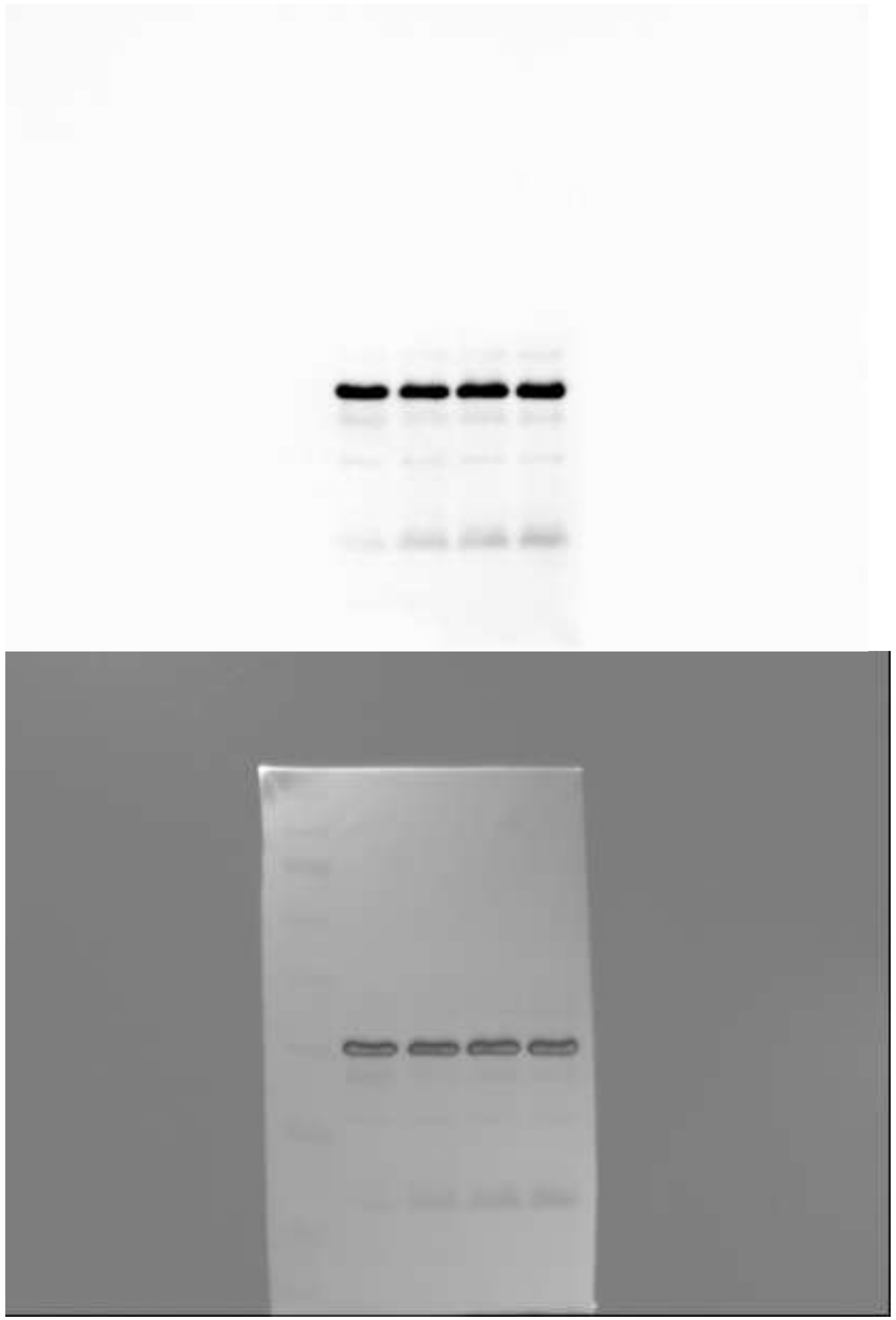

Figure 4. (H) XCT and GAPDH

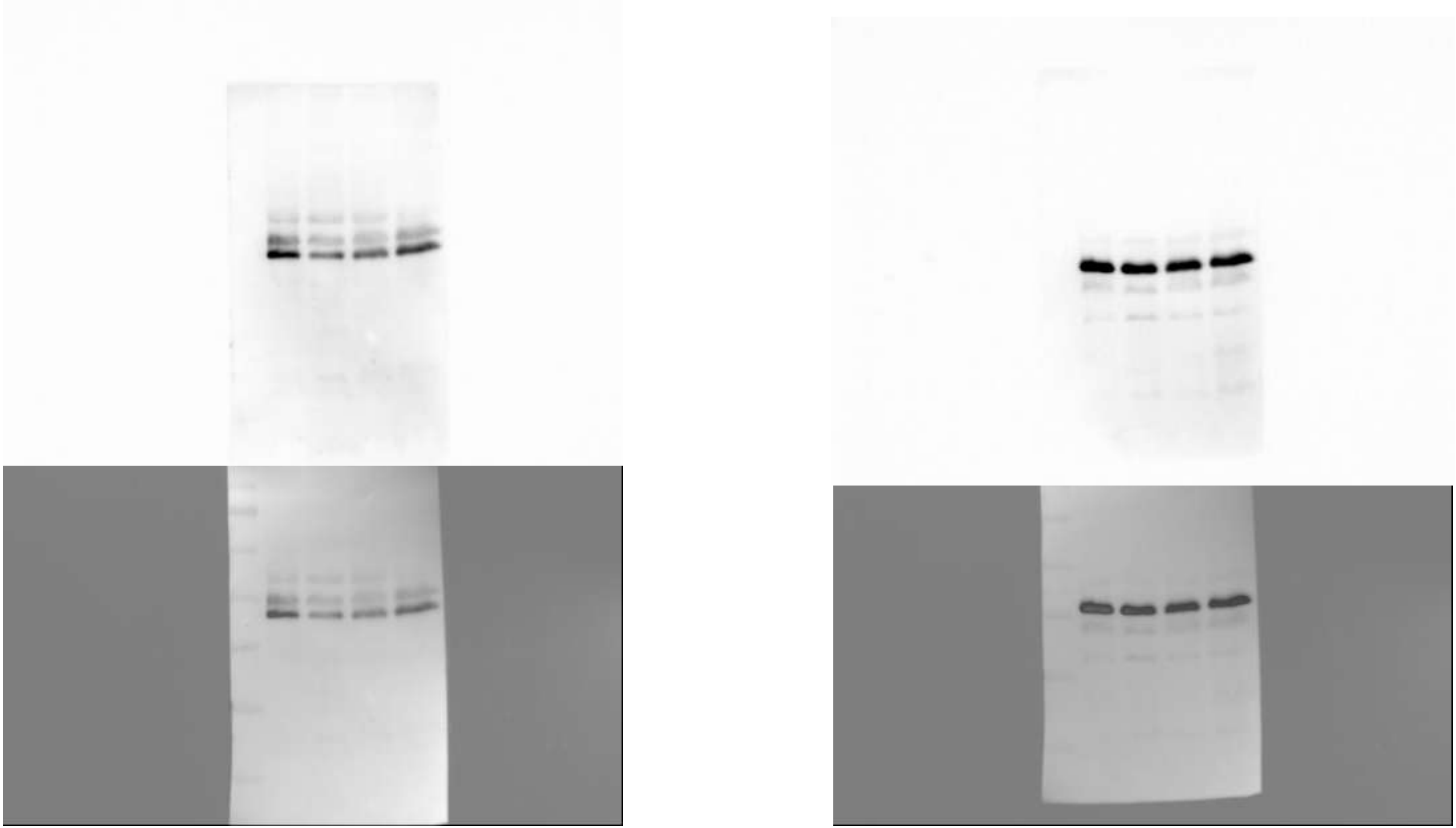

Figure 5. (A) BAX and GAPDH

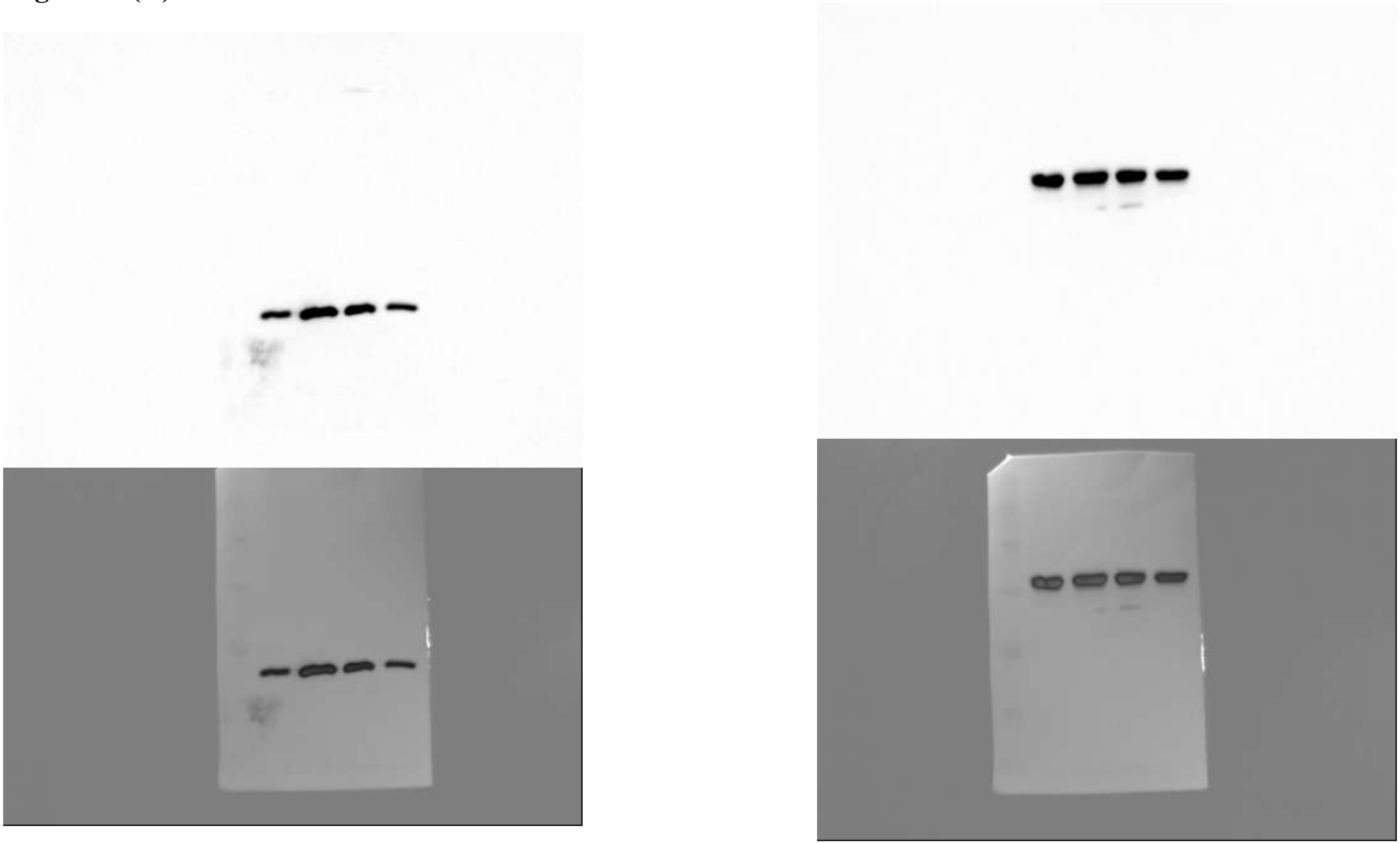

Figure 5. (B) BCL2 and GAPDH

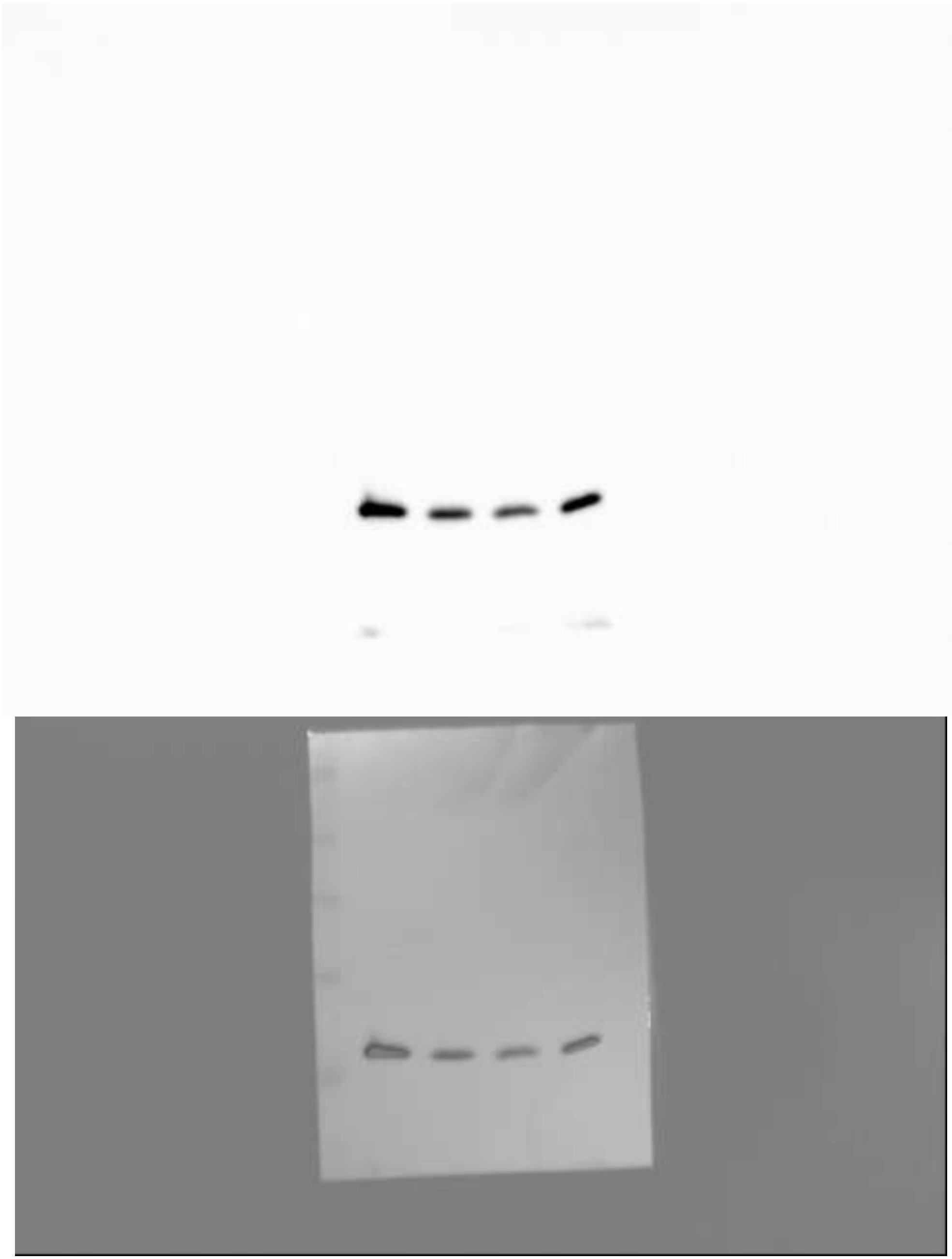

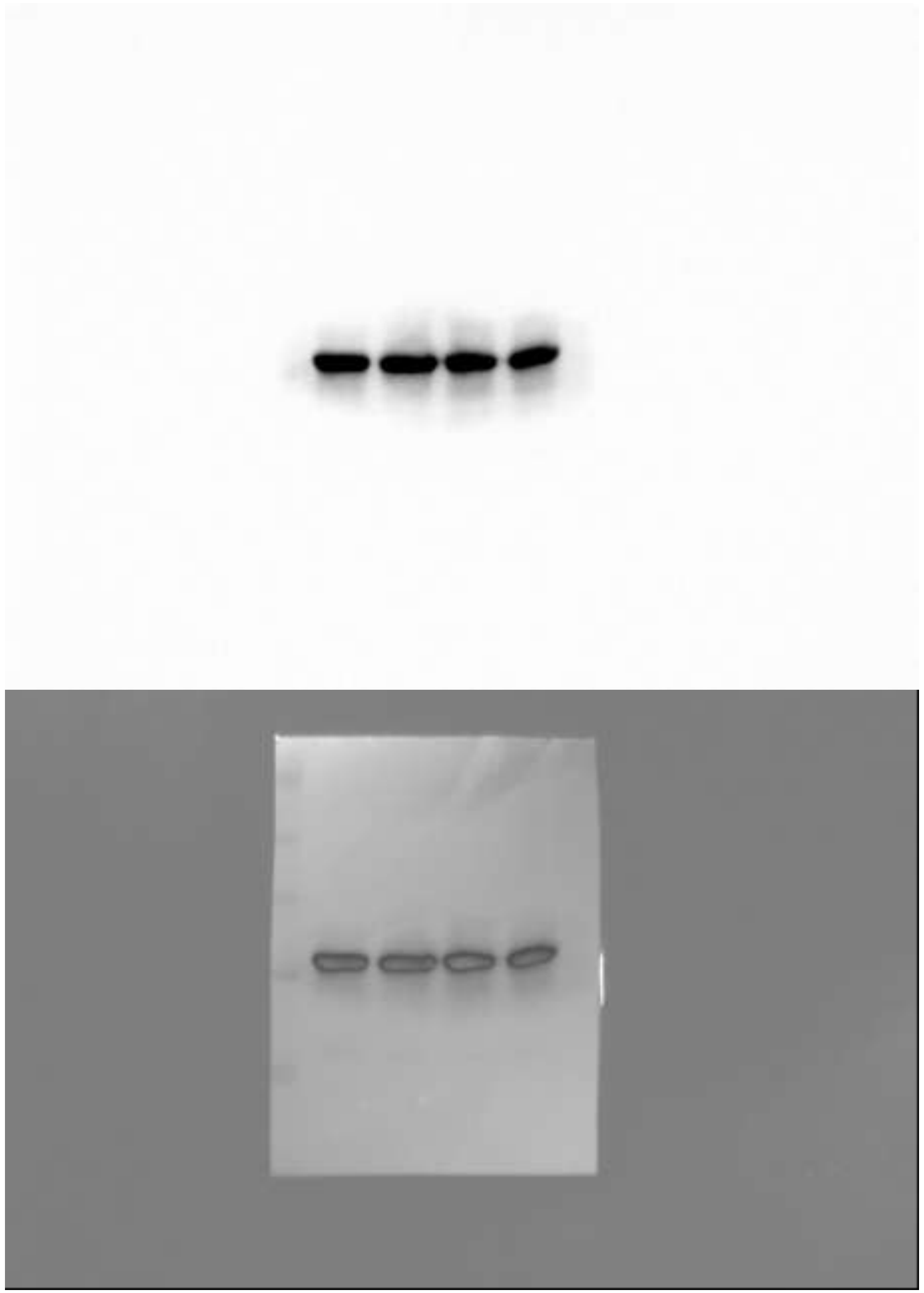

Figure 7. (A) MAPK3 and GAPDH

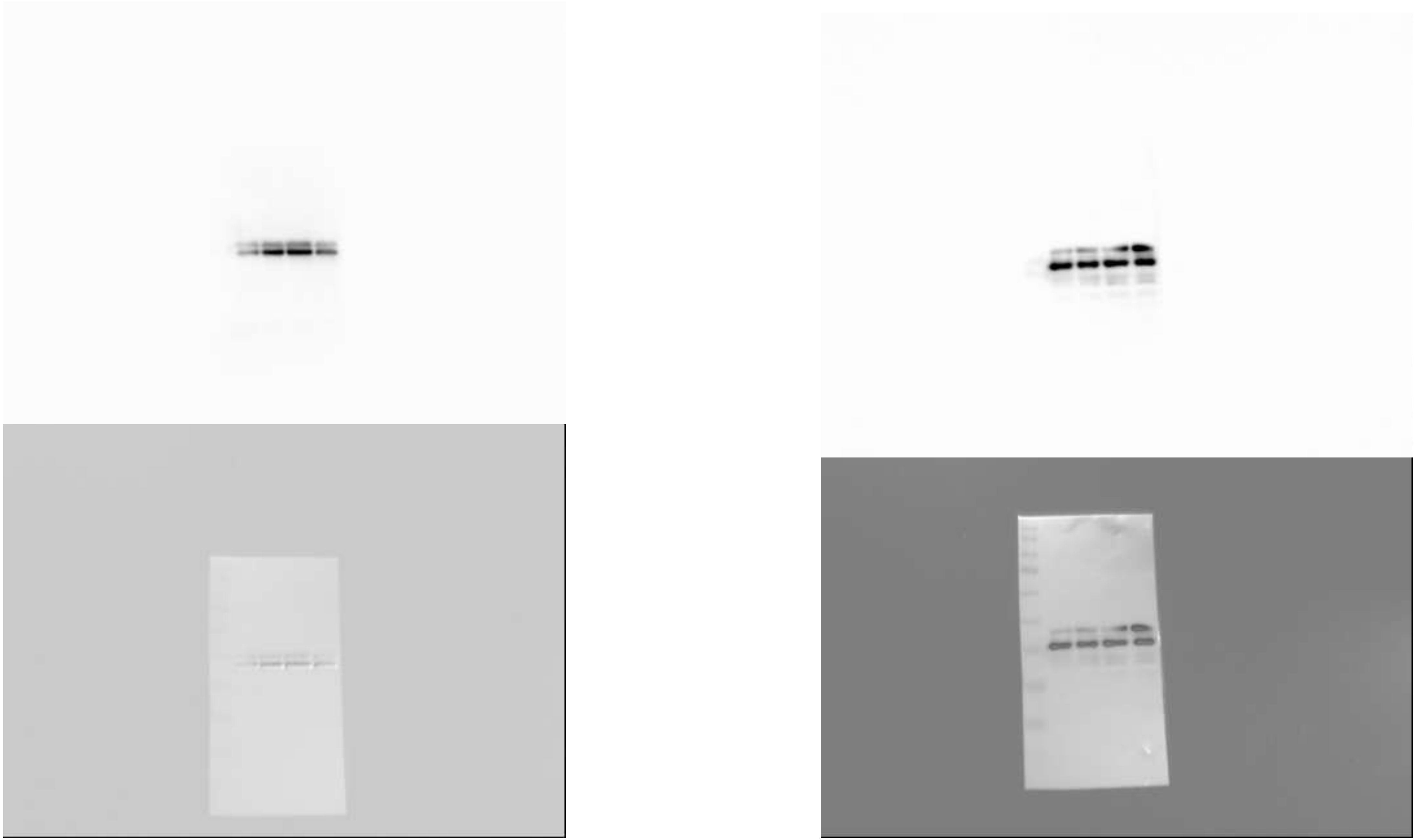

Figure 7. (B) CaMKII and GAPDH

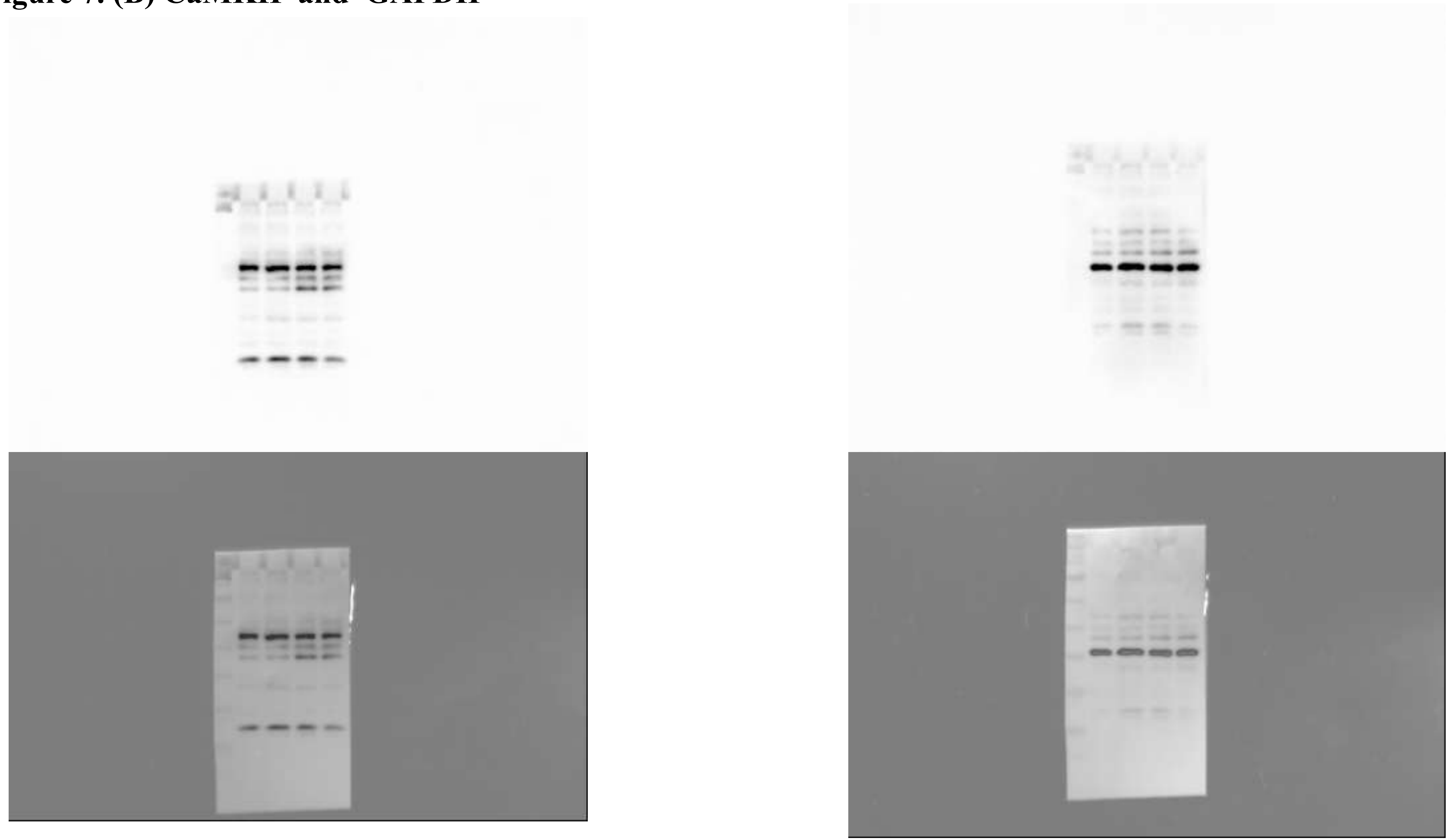

Figure 7. (C) p-CaMKII and GAPDH

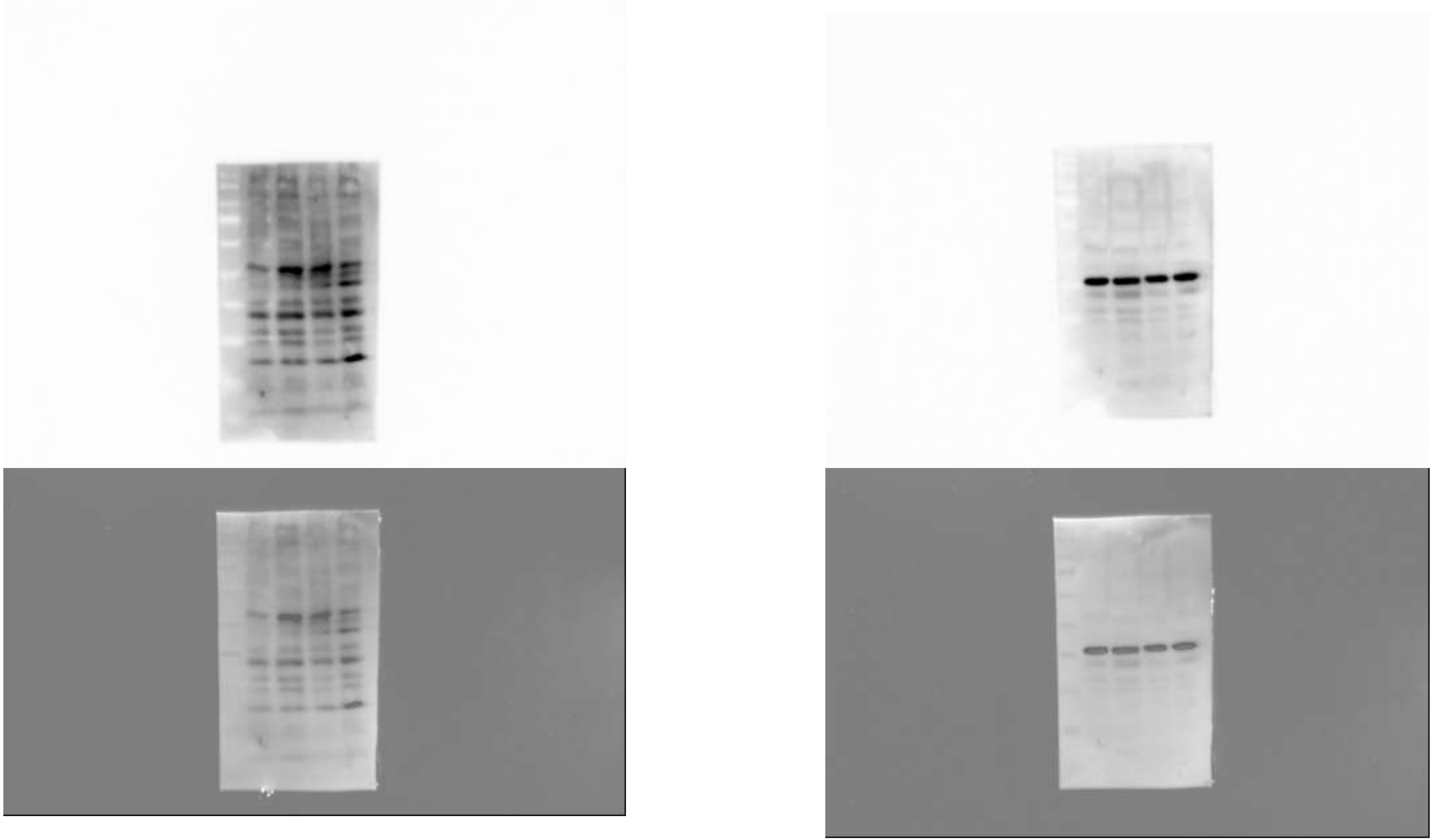

Figure 8. (A) ACSL4 and GAPDH

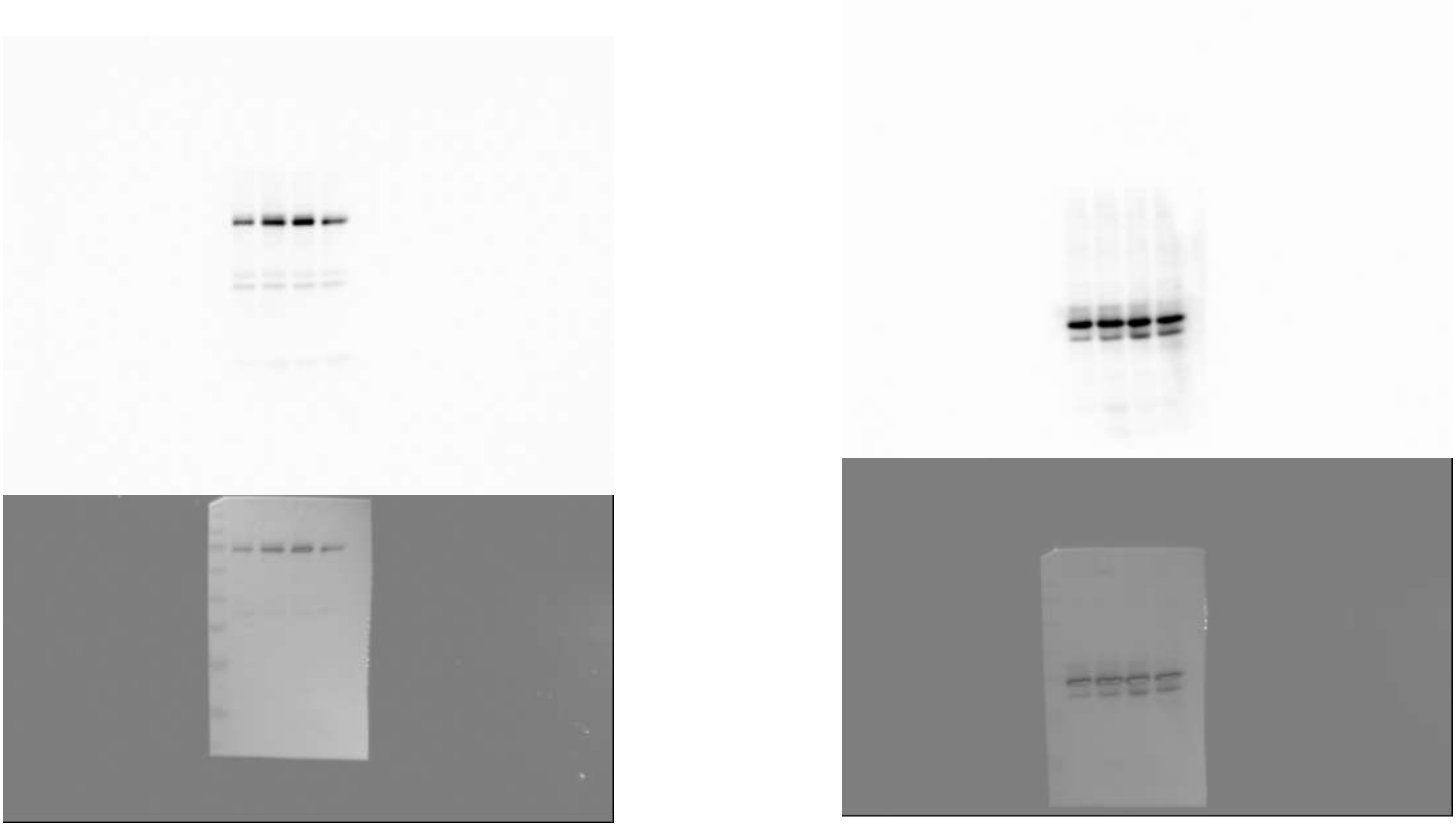

Figure 8. (B) Keap1 and GAPDH

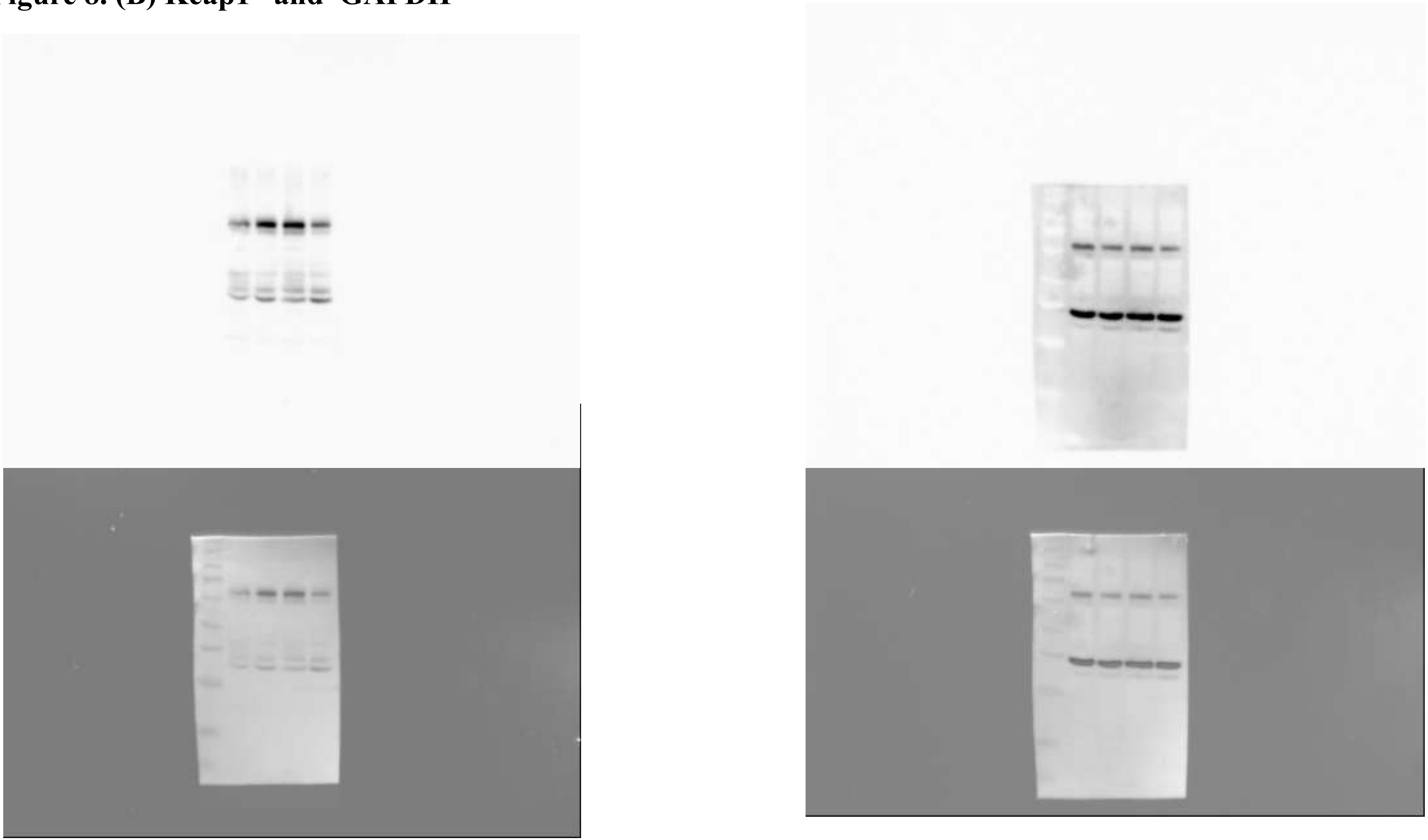

Figure 8. (C) NCOA4 and GAPDH

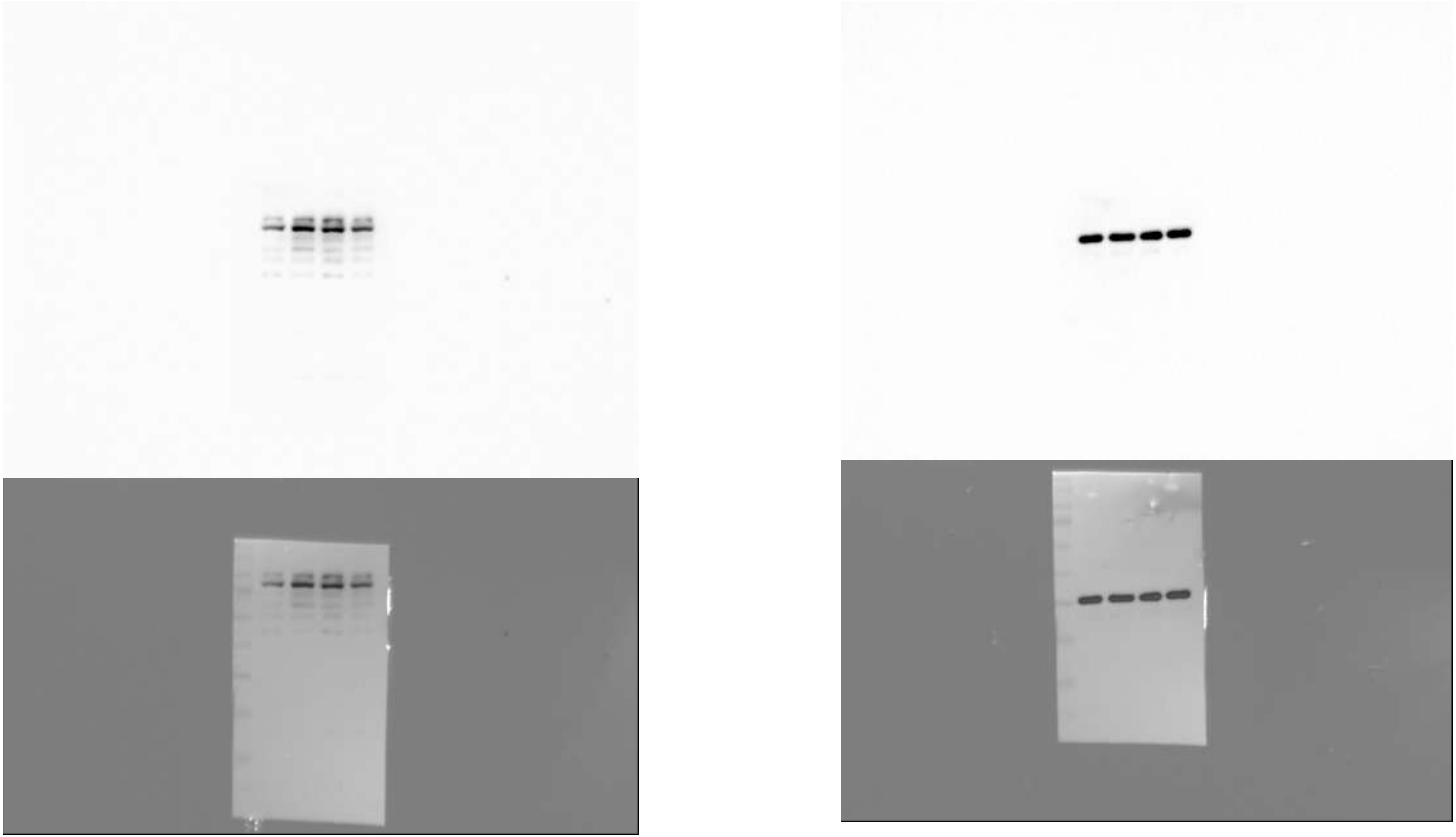

Figure 8. (D) GPX4 and GAPDH

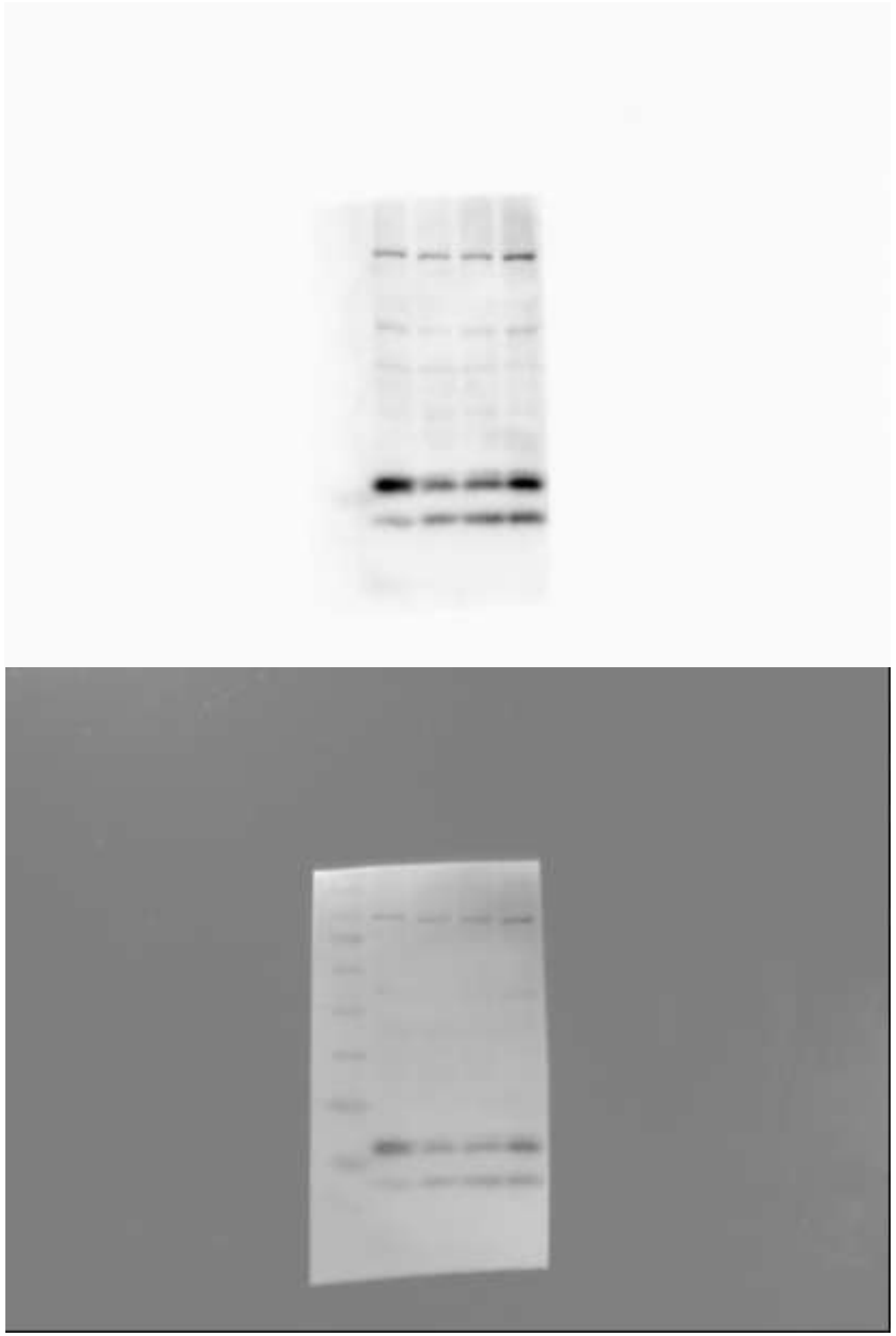

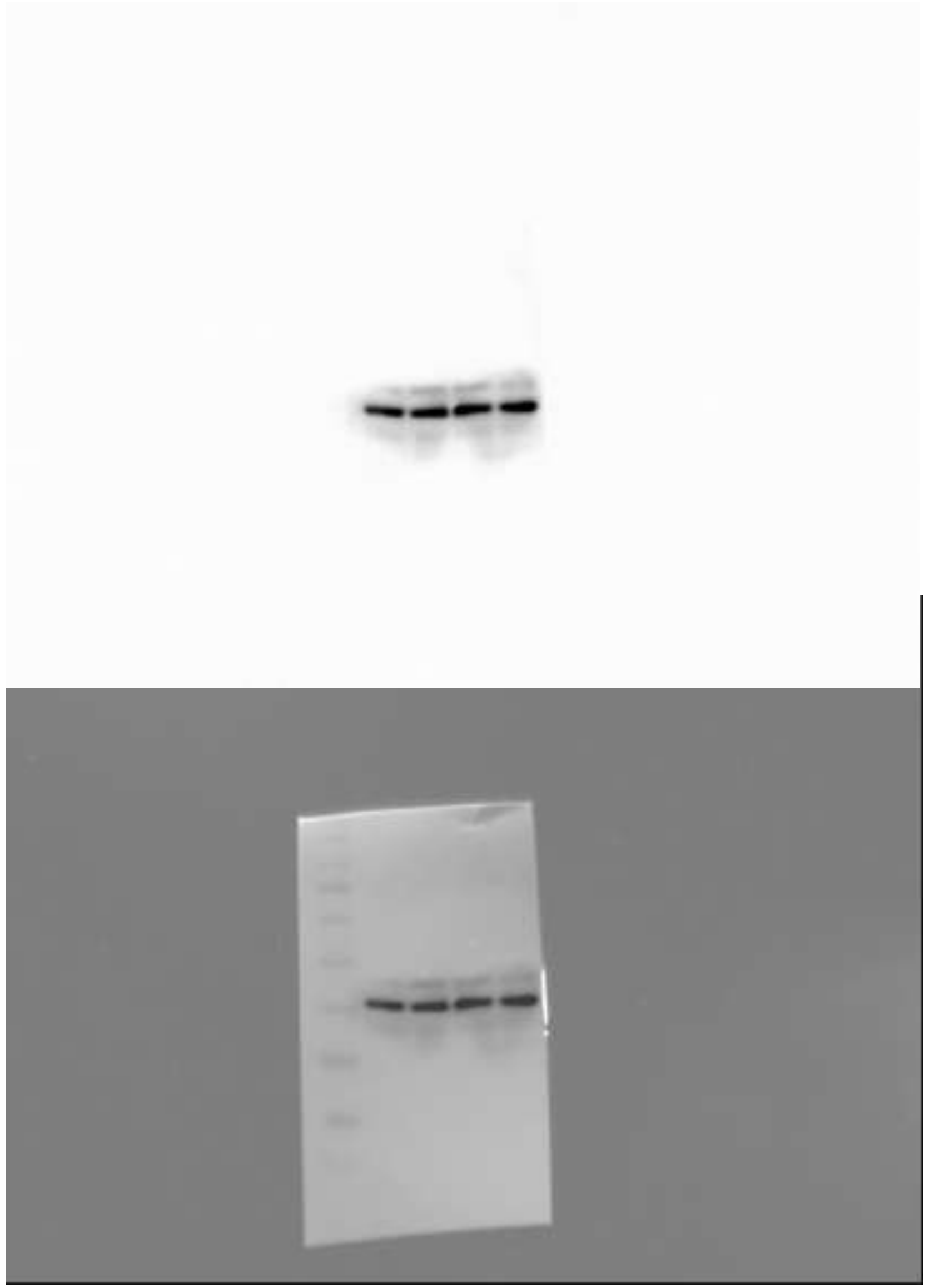

Figure 8. (E) NRF2   and GAPDH

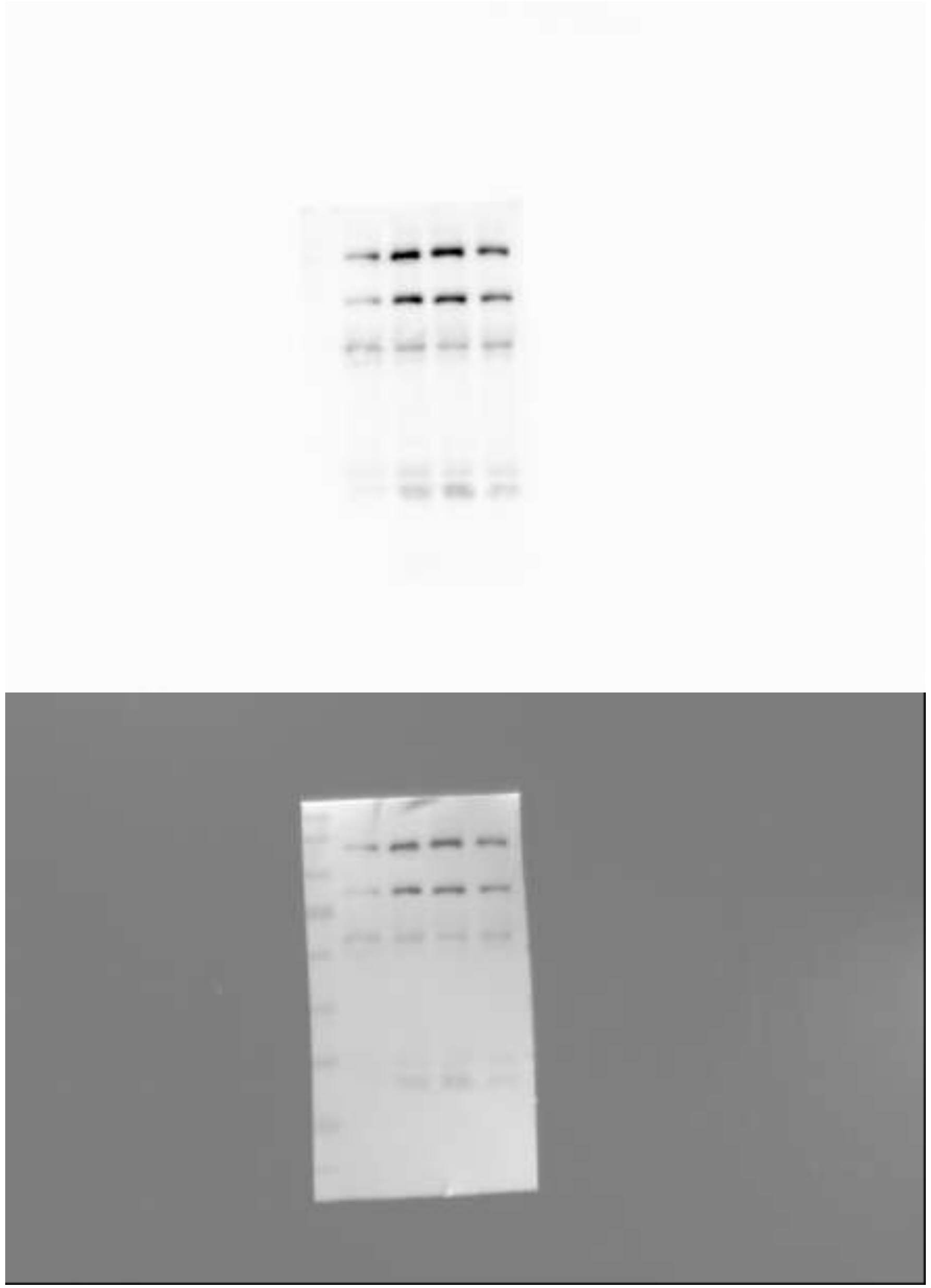

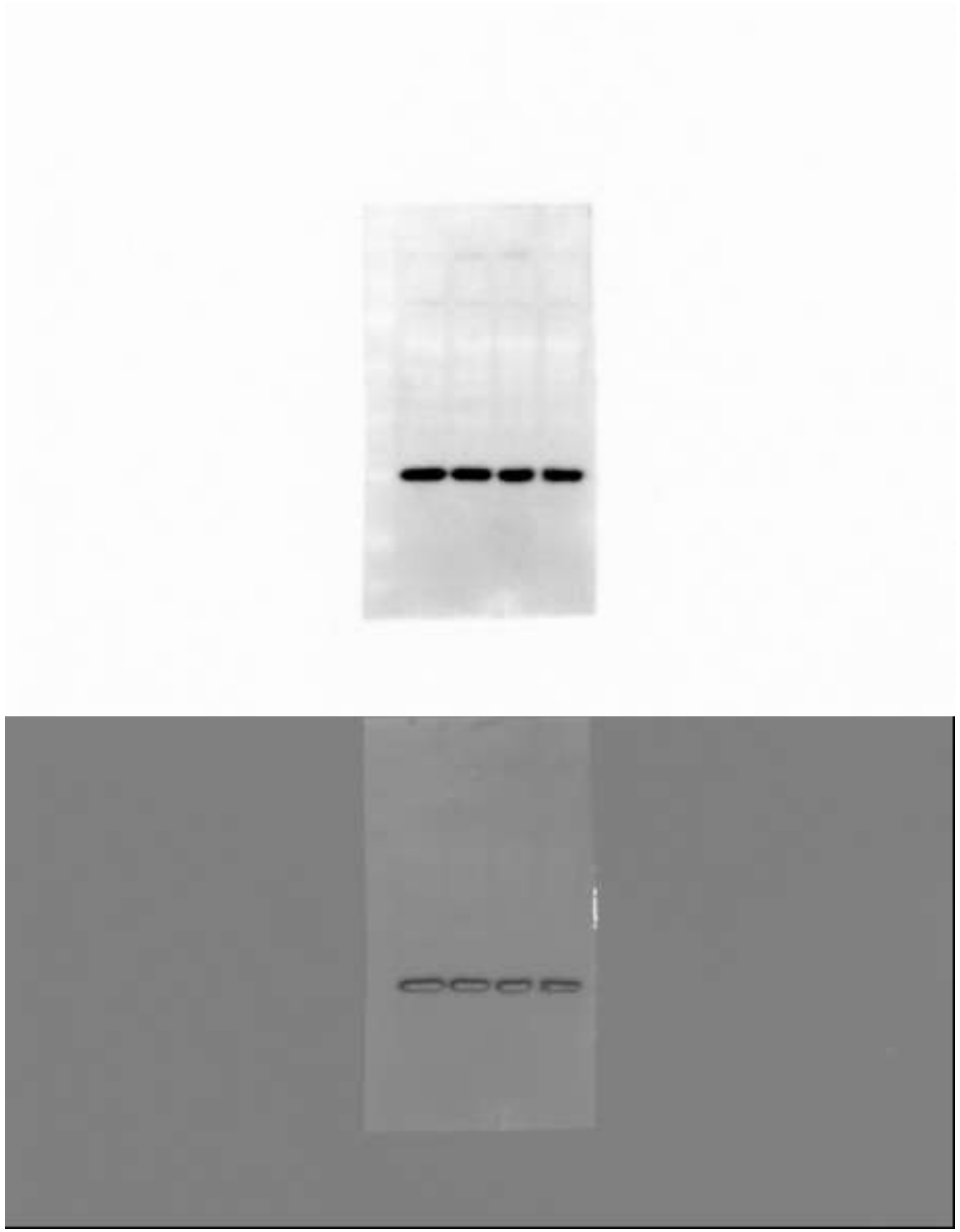

Figure 8. (F) HO-1 and GAPDH

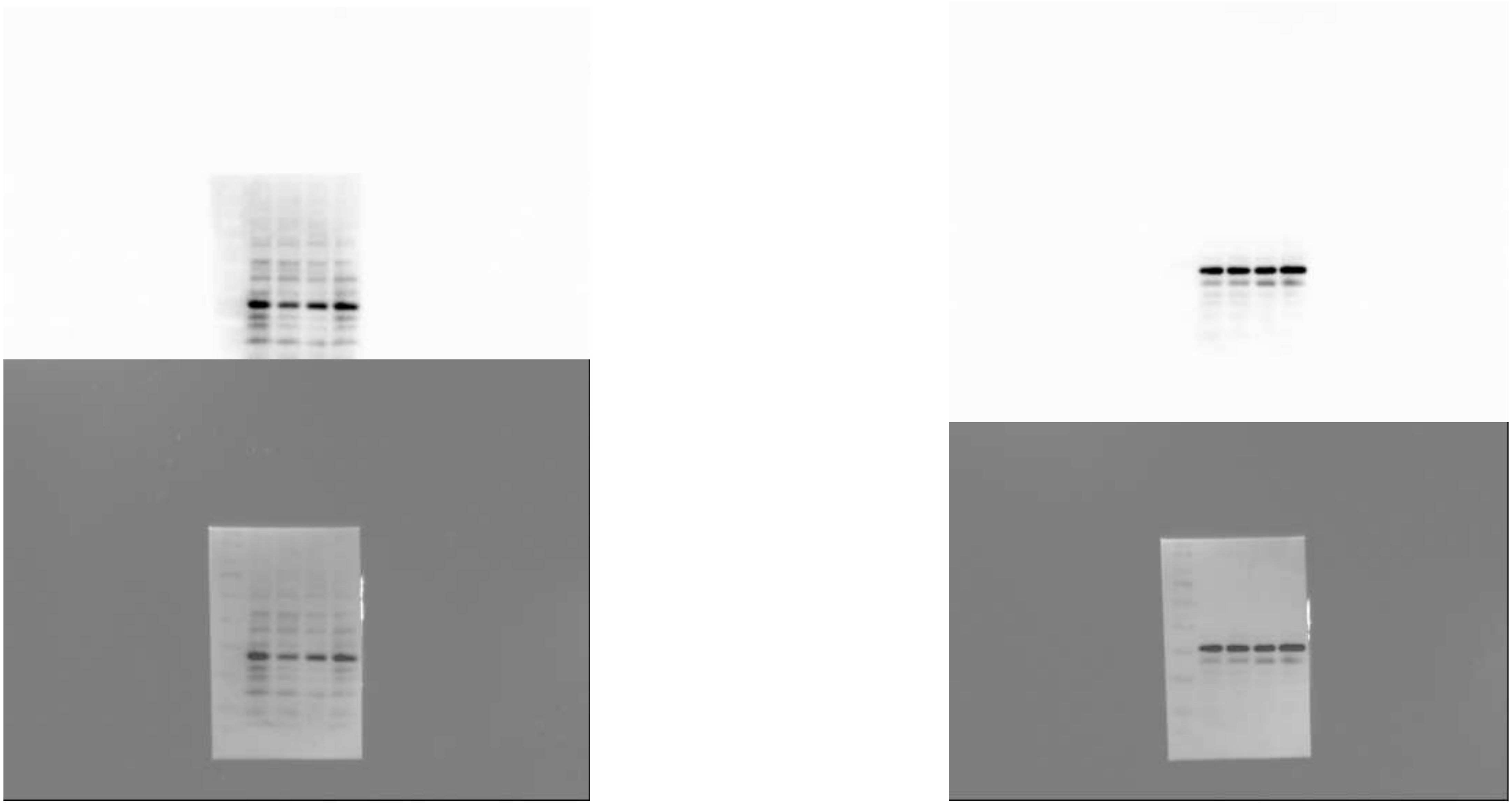

Figure 8. (G) FTH1 and GAPDH

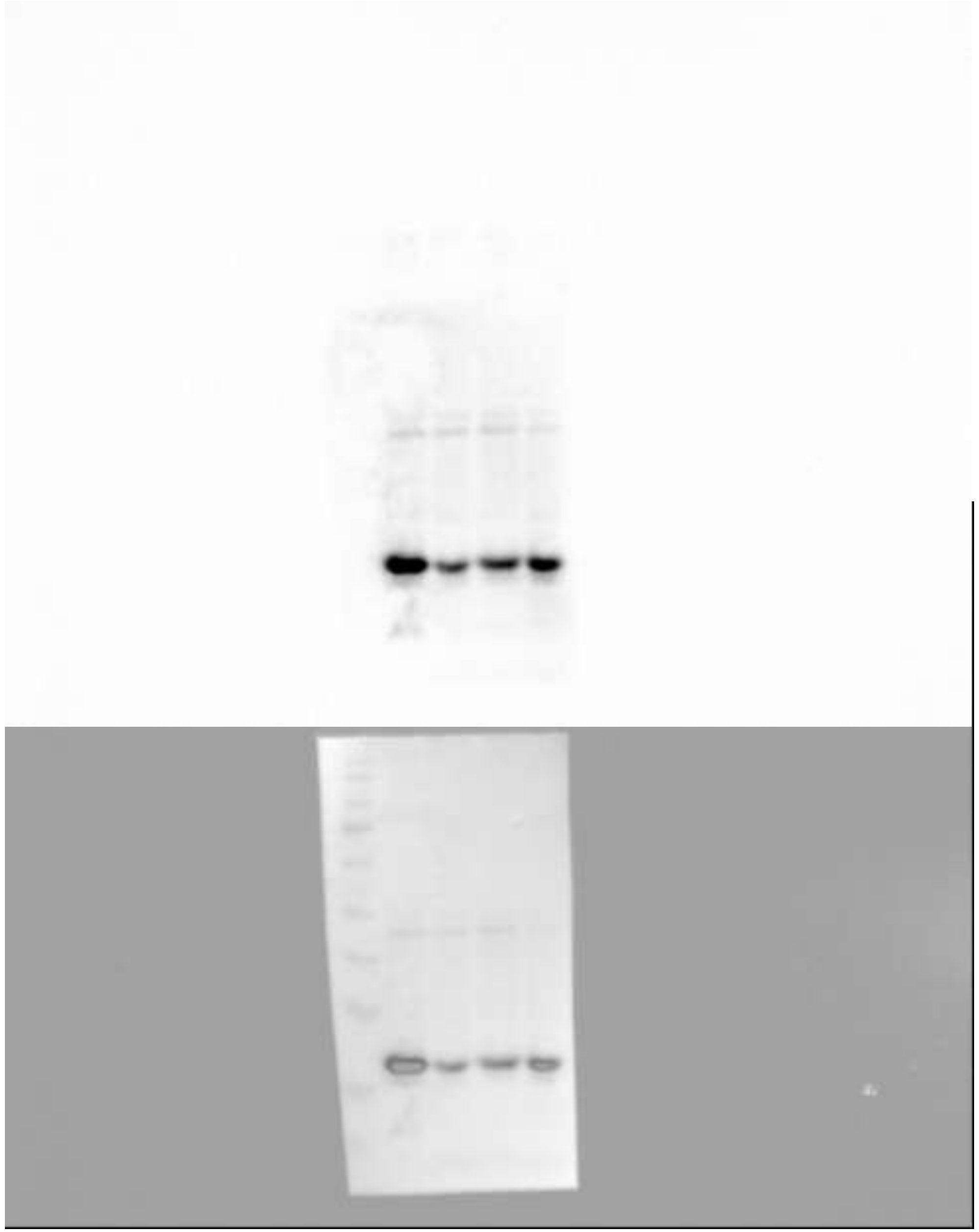

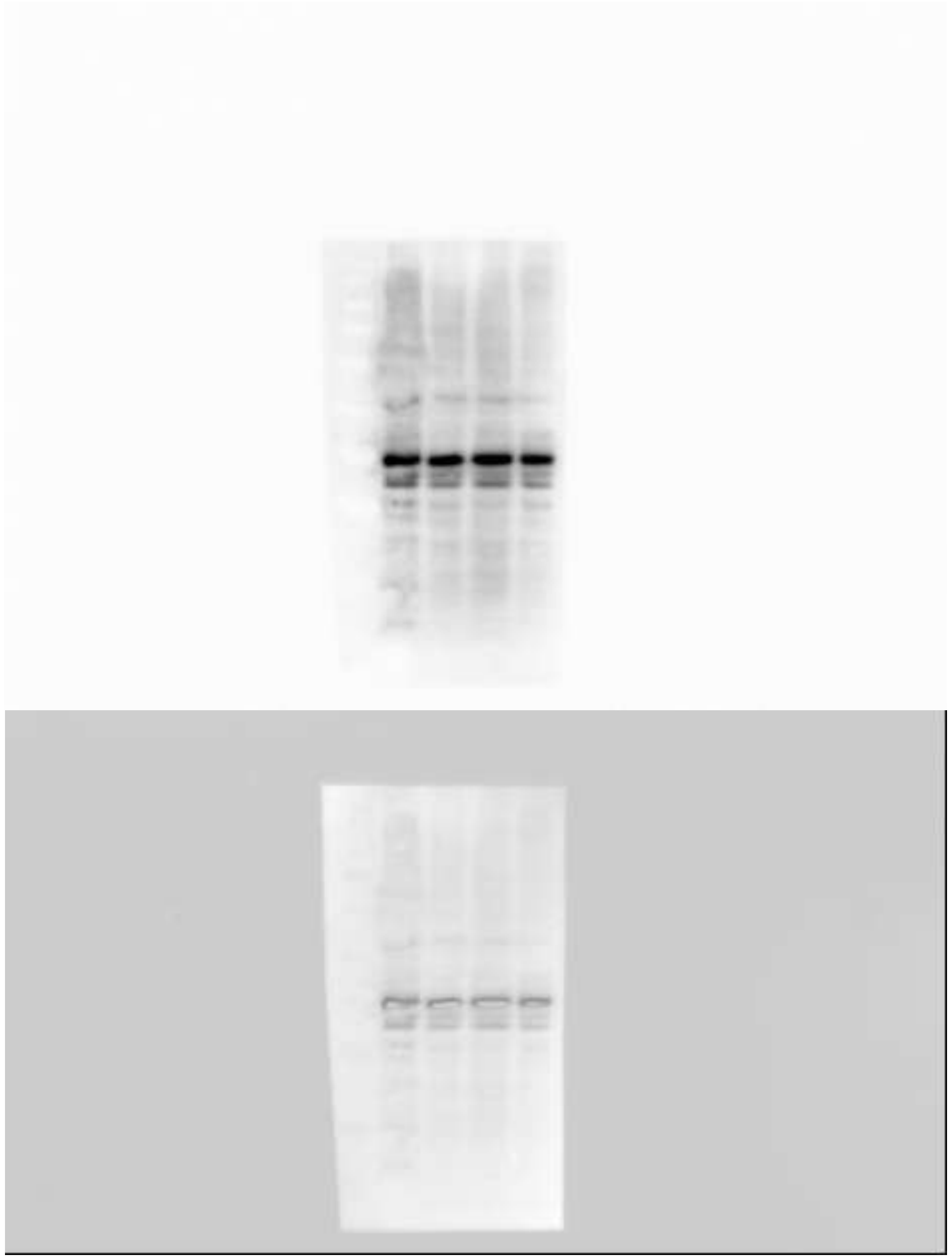

Figure 8. (H) XCT and GAPDH

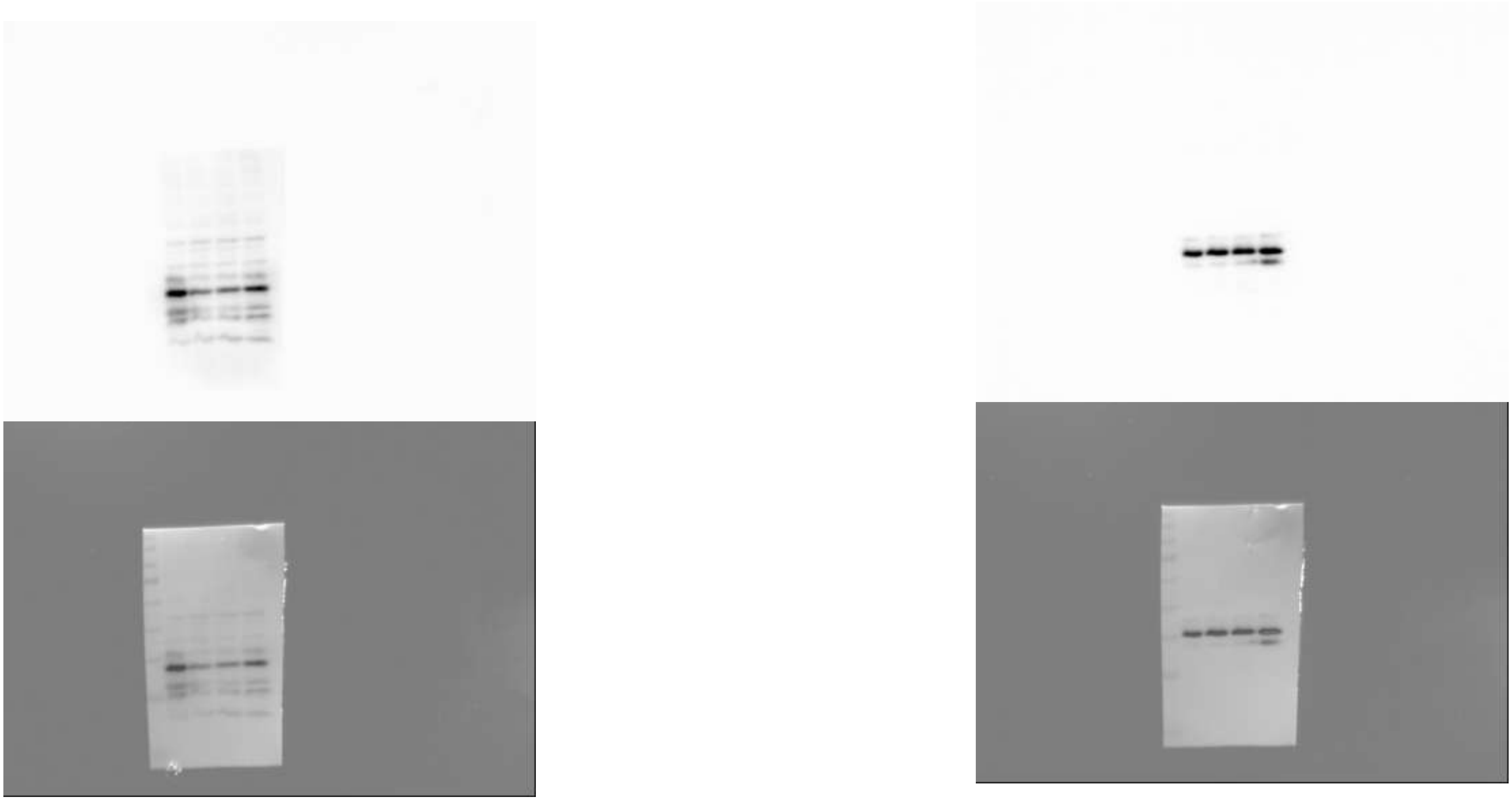

Figure 9. (A) BAX and GAPDH

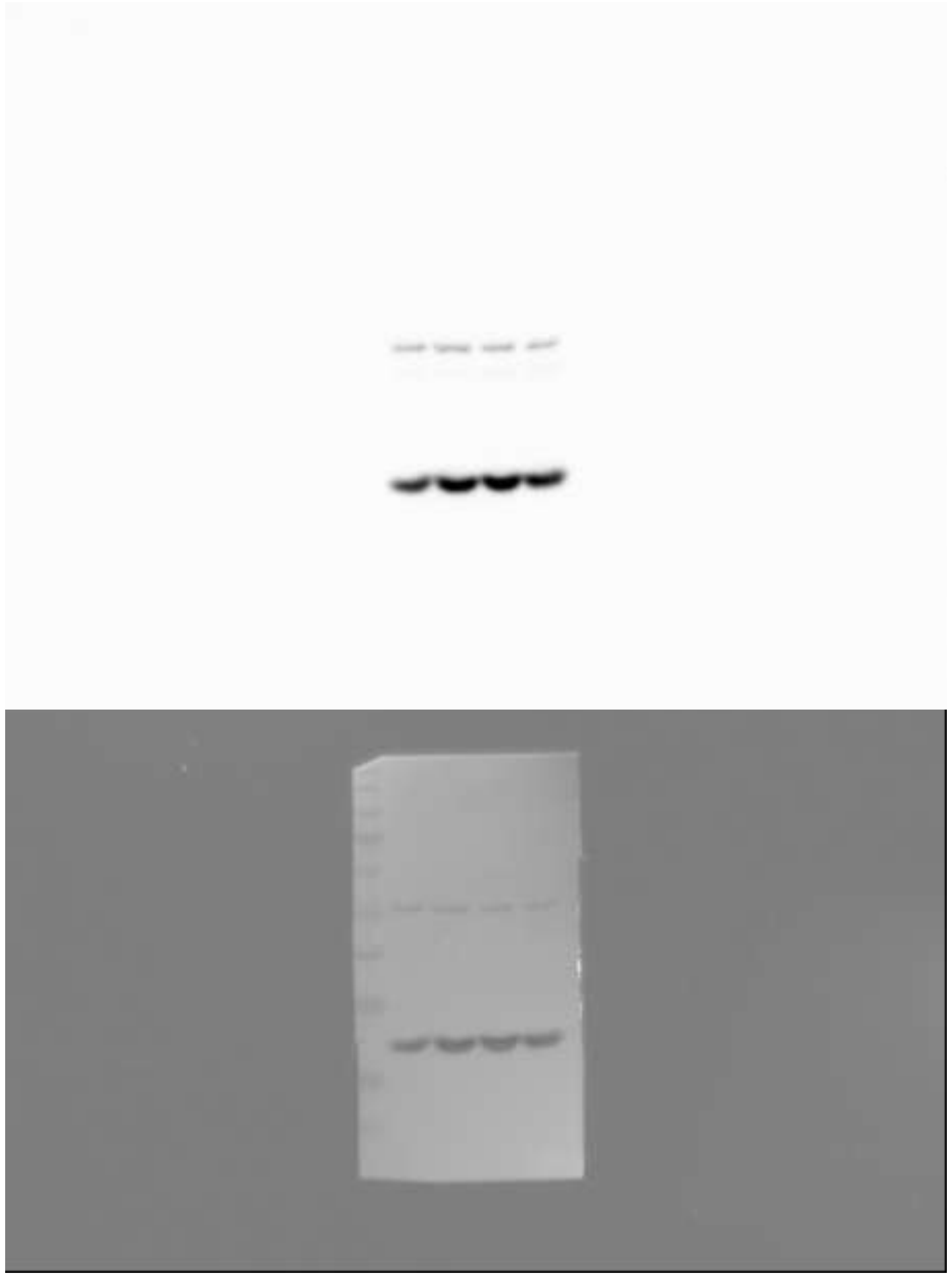

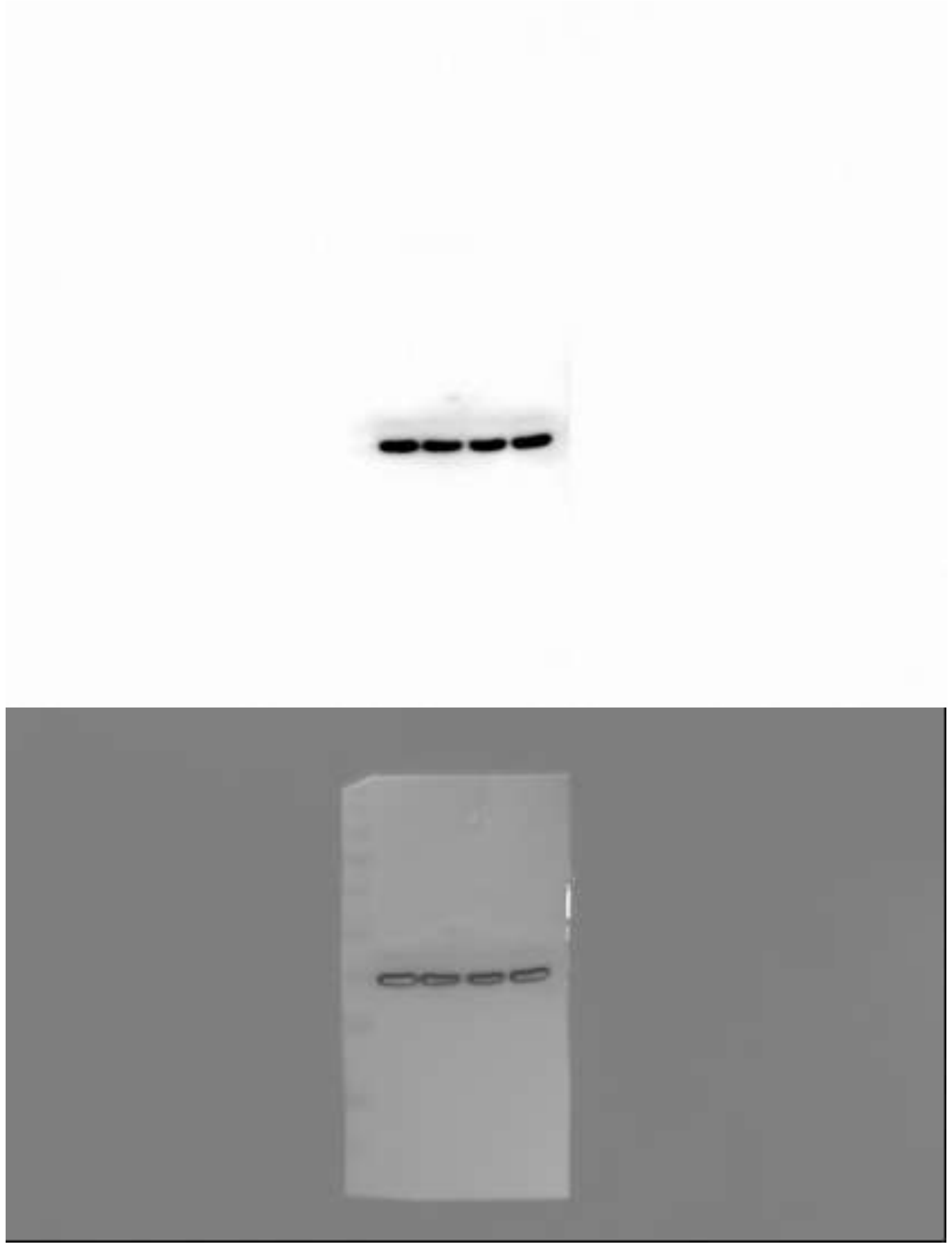

Figure 9. (B) BCL2 and GAPDH

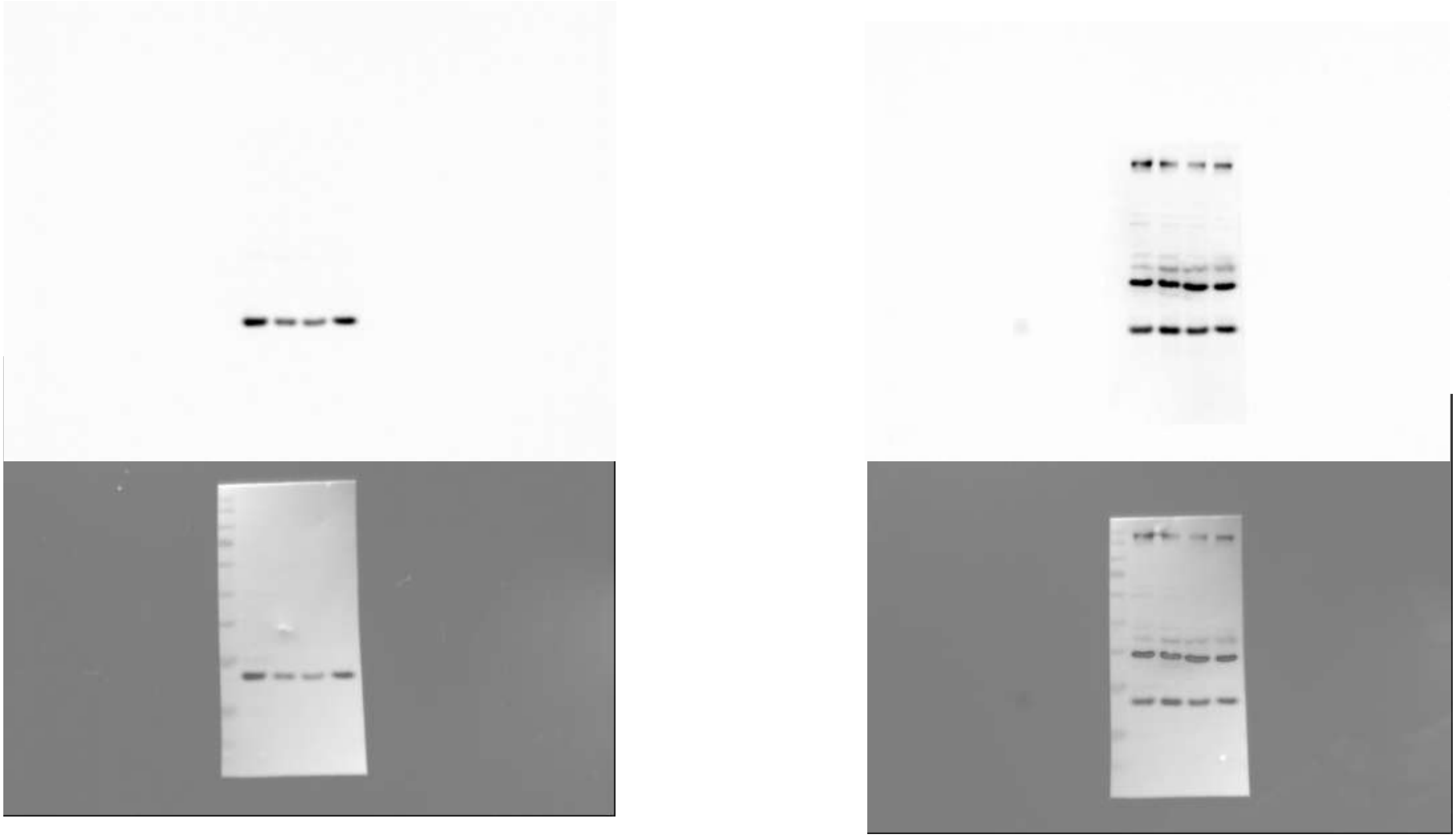

Supplement: Supplementary file 1 [file Data_Sheet_1.pdf]
